# Effect of fatty acid profiles in varying recipes of ready-to-use therapeutic foods on neurodevelopmental and clinical outcomes of children (6–59 months) with severe wasting: a systematic review

**DOI:** 10.1093/nutrit/nuad151

**Published:** 2023-12-22

**Authors:** Arista Nienaber, Cornelia Conradie, Geoffrey Manda, Bernadette Chimera-Khombe, Ettienne Nel, Edith B Milanzi, Robin C Dolman-Macleod, Martani J Lombard

**Affiliations:** Centre of Excellence for Nutrition (CEN), North-West University, Potchefstroom Campus, Potchefstroom, South Africa; Centre of Excellence for Nutrition (CEN), North-West University, Potchefstroom Campus, Potchefstroom, South Africa; Global Health Institute, Faculty of Medicine and Health Sciences, University of Antwerp, Antwerp, Belgium; Interdisciplinaire Sciences Santé, Universite Claude Bernard Lyon 1, Lyon, France; Department of Paediatrics and Child Health, University Stellenbosch, Bellville, Cape Town, South Africa; Medical Research Council Clinical Trials, University College London, London, United Kingdom; Centre of Excellence for Nutrition (CEN), North-West University, Potchefstroom Campus, Potchefstroom, South Africa; Centre of Excellence for Nutrition (CEN), North-West University, Potchefstroom Campus, Potchefstroom, South Africa

**Keywords:** docosahexaenoic acid, fatty acids, malnutrition, ready-to-use therapeutic foods, neurodevelopment, severe acute, wasting

## Abstract

**Context:**

In 2020, 13.6 million children under 5 years suffered from severe acute malnutrition (SAM)/wasting. Standard ready-to-use therapeutic foods (RUTFs) improve polyunsaturated fatty acid (PUFA) status but contain suboptimal amounts of omega-3 (n-3) PUFAs with unbalanced n-6-to-n-3 PUFA ratios.

**Objectives:**

The aim was to compare the effects of RUTFs with different essential fatty acid contents on PUFA status, neurodevelopmental, and clinical outcomes (mortality, comorbidities, and recovery) of children with severe wasting.

**Data Sources:**

Twelve databases, trial repositories, and article references with no publication limitations.

**Data Extraction:**

Ten studies from randomized, quasi, and cluster-randomized controlled trials providing RUTFs as home treatment to children 6–59 months with SAM/wasting were included.

**Data Analysis:**

Plasma phospholipid eicosapentaenoic acid content was higher in children receiving RUTF with altered essential fatty acid contents compared with standard RUTF (0.20 [0.15–0.25], P < 0.00001). Docosahexaenoic acid (DHA) status only improved in children receiving RUTF with added fish oil (0.33 [0.15–0.50], P = 0.0003). The Malawi Developmental Assessment tool (MDAT) global development and problem-solving assessment scores were higher in global assessment and gross motor domains in children receiving added fish oil compared with standard formulation (0.19 [0.0–0.38] and 0.29 [0.03–0.55], respectively). Children receiving high-oleic-acid RUTF (lowering the n-6:n-3 PUFA ratio of the RUTF) with or without fish oil had significantly higher scores in social domains compared with those receiving the standard formulation (0.16 [0.00–0.31] and 0.24 [0.09–0.40]). Significantly higher mortality risk was found in children receiving a standard formulation compared with RUTF with a lower n-6:n-3 PUFA ratio (0.79 [0.67–0.94], P = 0.008).

**Conclusion:**

Although lowering n-6:n-3 PUFA ratios did not increase plasma DHA, it improved specific neurodevelopmental scores and mortality due to lower linoleic acid (high-oleic-acid peanuts), higher alpha-linolenic acid (altered oil), or both. Additional preformed n-3 long-chain PUFAs (fish oil) with RUTF improved the children’s DHA status, neurodevelopmental outcomes, and weight-for-height z score. More research is needed regarding cost, availability, stability, acceptability, and the appropriate amount of n-3 long-chain PUFAs required in RUTFs for the best clinical outcomes.

**Systematic Review Registration:**

PROSPERO registration no. CRD42022303694.

## INTRODUCTION

Severe wasting, a condition where children are too thin for their height, is indicative of severe acute malnutrition (SAM). Severe wasting is diagnosed by a weight-for-height *z* score (WHZ) less than –3 standard deviations (SDs) and/or a midupper arm circumference (MUAC) less than 115 mm with or without the presence of bipedal nutritional edema.^1^ It is estimated that, in 2020, approximately 45.4 million children under the age of 5 years suffered from wasting (WHZ < –2), of whom 13.6 million suffered from severe wasting (WHZ < –3).^2^

Fat malabsorption has been reported in over 50% of children with SAM.^3–5^ The severity of fat malabsorption is associated with the degree of protein deficiency and is further attributed to intestinal bacterial overgrowth and diarrhea.^3^^,^^6^^,^^7^ Due to fat malabsorption and insufficient intakes of essential fatty acids (EFAs), children with SAM present with poor polyunsaturated fatty acid (PUFA) status, especially omega-3 long-chain PUFAs (n-3 LCPUFAs), compared with their healthy counterparts.^8–16^ n-3 LCPUFAs (ie, PUFAs with ≥18–20 carbon atoms, above all, docosahexaenoic acid [DHA; 22:6n-3]), have important functions in neural development, brain function, and vision.^17–22^ DHA modulates neuronal signal transduction, gene expression, and neuronal membrane physical properties, thereby playing an irreplaceable role in neural development during pregnancy, lactation, and the first years of life.^23^ Deprivation of n-3 LCPUFAs during early childhood leads to lower n-3 PUFA plasma status together with the replacement of DHA in the brain by n-6 docosapentaenoic acid (22:5n-6). This has been reported to result in neurocognitive deficits, including aggression, as well as problems with impulse control, balance, learning, and behavior.^24^^,^^25^ Depending on the duration and critical developmental period, low n-3 LCPUFA brain status can cause long-term neurodevelopmental impairments. Various studies indicated that SAM survivors have memory and visual attention impairments 7 years after SAM treatment.^26–30^ In addition to the essential functions in neurodevelopment, implications of unfavorable LCPUFA profiles have been recognized concerning anthropometric measures and growth, hemoglobin concentrations, blood clotting, pain sensitivity, immune responses, and inflammatory outcomes.^15^^,^^16^^,^^31–34^

Ready-to-use therapeutic foods (RUTFs) are the principal home-based nutritional treatment for uncomplicated severe wasting.^35^^,^^36^ RUTFs serve as the sole food source during the treatment period of 8 to 12 weeks. The nutritional composition, including PUFA content, of RUTFs must, therefore, be complete and suitable to support optimal child growth and development.^1^^,^^37^^,^^38^

Dietary linoleic acid (LA; 18:2n-6) and alpha-linolenic acid (ALA; 18:3n-3) are EFAs consumed from the seeds, nuts, and oils of different plants (eg, canola and flaxseed). The fatty acids can be incorporated into various tissues and/or elongated and desaturated to LCPUFAs. Enzymatic reactions occurring mainly in the liver, but to a lesser degree also in other tissues such as the brain and kidneys, synthesize LCPUFAs from LA and ALA.^39^^,^^40^ These enzymatic reactions include the elongation, by adding two-carbon units, and desaturation, by the addition of double bonds to produce arachidonic acid (20:4n-6), eicosapentaenoic acid (EPA; 20:5n-3) and DHA from LA and ALA, respectively.^40^^,^^41^ The desaturase activity and synthesis rates of LCPUFAs differ between the different tissues and are reliant on various factors such as the protein concentration of the enzymes involved, genetic factors, age and dietary intake, and status of fatty acids.^40^^,^^41^

Hsieh et al^42^ reported healthy desaturase activity in children with SAM; hence, these children can synthesize EPA and DHA when provided RUTFs with appropriate LA:ALA ratios (between 1:1 and 5:1). However, commonly used standard RUTF, which is primarily peanut butter and milk powder–based, may contain an unbalanced LA:ALA ratio (as high as 53:1).^37^ Since the metabolism of n-6 and n-3 PUFAs use the same enzymes for desaturation, high LA:ALA ratios in diets suppress the endogenous production of n-3 LCPUFAs, including DHA.^8^^,^^40^ Evidence suggests that RUTFs in malnourished children improve EFA deficiency but do not provide sufficient ALA, EPA, or DHA for optimal LCPUFA status to support neural development and other important functions.^14^ Moreover, treating children diagnosed with SAM, and therefore severe wasting, with standard RUTFs results in lower plasma phospholipid DHA status (–25%).^42^^,^^43^

To address this problem, RUTFs with a modified fatty acid profile by either the addition of (1) ALA, (2) n-3 LCPUFAs, or (3) the lowering of LA content have been investigated. Alternative RUTF recipes designed to lower costs and improve the availability and shelf-life of RUTFs (eg, higher soy content) have also been included in the investigation. This systematic review and meta-analysis therefore aimed to compare the effects of RUTFs with different EFA contents on outcomes such as neurodevelopment, mortality, comorbidities, and nutritional recovery in children diagnosed with severe wasting to provide evidence-based information for policy development. RUTFs with an alternative recipe that unintentionally altered the fatty acid content were also included in this systematic review to investigate how changes in fatty acid content, irrespective of whether it favored higher or lower LA:ALA ratios, alter these outcomes.

## METHODS

### Protocol and registration

A protocol for this systematic review was registered on the International Prospective Register of Systematic Reviews (PROSPERO CRD42022303694). The Preferred Reporting Items for Systematic Review and Meta-Analyses (PRISMA) guidelines were followed in reporting this review.

### Information source

A comprehensive systematic literature search was performed to identify relevant studies, with no limitations regarding language, geographical area, date of publication, and publication status. The following electronic databases were searched for electronic sources up to May 1, 2023: EMBASE, MEDLINE, Scopus, Science Direct, Web of Science, CINAHL, LILACS, Cochrane Database of Systematic Reviews, and Cochrane Central Register of Controlled Trials. Unpublished and ongoing trials were identified by searching clinical trial repositories, such as the National Institutes of Health Clinical Trials Register (http://clinicaltrials.gov), International Standard Randomized Controlled Trials Number (ISRCTN) registry, and Cochrane Central Register of Controlled Trials. Conference abstracts and proceedings were considered by searching BIOSIS Previews (EBSCO).

### Search strategy and study selection

**Figure 1:**
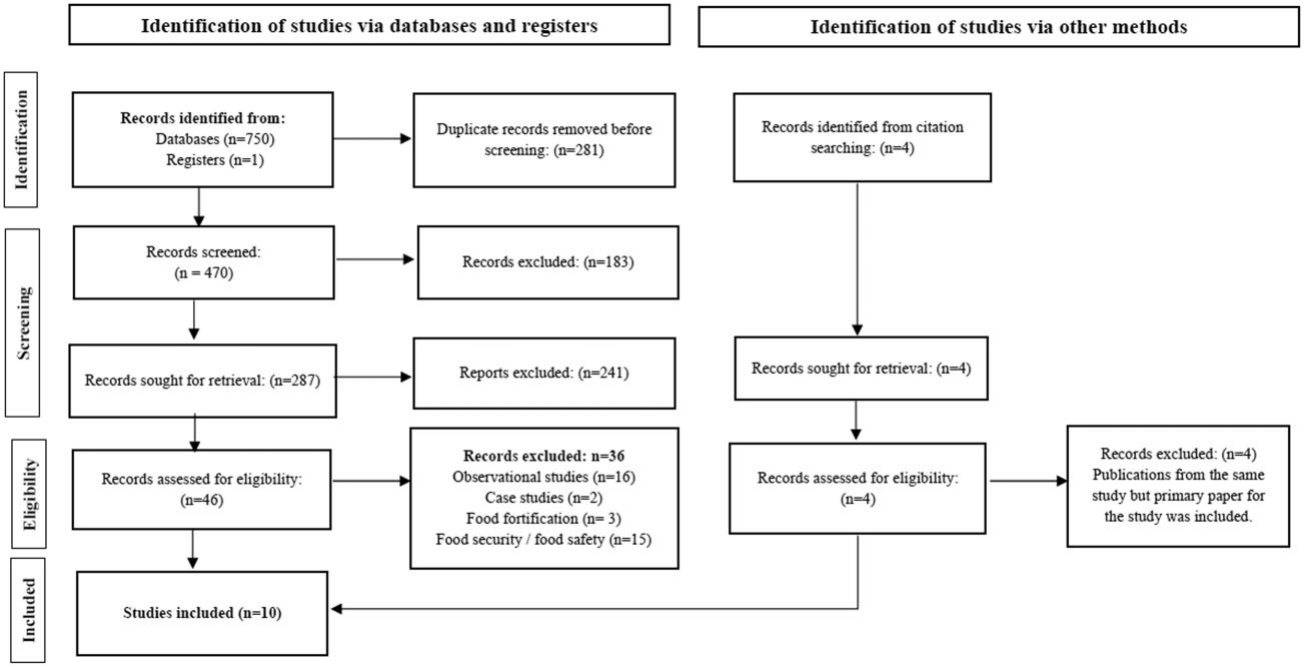
Flow diagram of the literature search process.

The PICOS (Participants, Interventions, Comparators, Outcomes, and Study design) criteria that were used to define the research question are shown in Table 1. Table 2 shows the search strategy that was used. Corresponding authors of the trials included in the study and unpublished trials identified in the trial registries were contacted if additional information was required. Three review authors (A.N., C.C., and M.J.L.) independently determined the eligibility of studies. Studies were initially screened by titles and duplicates were excluded. After this, all potentially eligible articles were screened by abstract. Differences in opinion were discussed by the research team to reach an agreement on which studies to include for screening. Thereafter, the full articles were screened to ascertain eligibility with an eligibility form designed from the PICO. A flow diagram of article selection is shown in Fig. 1.

**Table 1:** PICOS criteria for inclusion of studies

Parameter	Criteria
Participants	Infants and children, aged 6 to 59 months, diagnosed with wasting (defined as WHZ < −2 SDs or MUAC < 125 mm) and/or edematous malnutrition. Participants with complicated acute malnutrition and with additional co-morbidities that required hospitalization were either excluded or only included after stabilization and discharge from the hospital.
Interventions	RUTF with essential fatty acid profiles different from current standard RUTF formulations, such as those with high-oleic-acid, low-linoleic-acid content (eg, high-oleic peanut, palm oil, and linseed oil); high-alpha-linolenic acid content (eg, flaxseed oil containing RUTFs with or without fish oil capsules); or with corn/soy blend oil–based content.
Comparators	The comparator was the current international standard of care RUTF formulations as recommended by the WHO.
Outcomes	To prevent misleading results, denominators for the outcomes were based on the intention-to-treat (ITT) analysis: Fatty acid status; Neurodevelopmental outcomes included: global development; fine motor development; gross motor development; language; social development; and cognition (eg, memory, attention, and visual functioning); Nutritional recovery included: the rate of weight gain; rate of length/height gain; rate of MUAC gain; anthropometrical status (WHZ, WAZ, HAZ); recovery (as defined by study authors); Comorbidities included fever, diarrhea, inflammation, infections, and rash; Adverse events as defined by the study author; Mortality; Acceptability, cost, and feasibility as exploratory outcomes.
Study design	RCTs, including quasi-randomized trials and cluster randomized trials. Open-label trials were included, provided that random allocation was applied. The potential bias introduced was addressed through sensitivity analyses.

*Abbreviations:* HAZ, height-for-age *z* score; MUAC, midupper arm circumference; RCT, randomized controlled trial; RUTF, ready-to-use therapeutic food; SD, standard deviation; WAZ, weight-for-age *z* score WHO, World Health Organization; WHZ, weight-for-height *z* score.

**Table 2:** Search strategy

No.	Search terms
1	(“infant”/exp OR “infant” OR “child”/exp OR “child” OR “preschool”/exp OR “preschool” OR toddler* OR preschool* OR kindergarten* OR “under 9s” OR “under 9” OR “under nine*” OR “paediatric” OR “paediatric” OR [under AND nine*]) AND “controlled study”/de AND ([child]/lim OR [preschool]/lim)
2	(nutrition AND disorder OR “nutrition disorders”/exp OR “nutrition disorders” OR “protein energy malnutrition”/exp OR “protein energy malnutrition” OR “undernutrition”/exp OR undernutrition OR “under nutrition” OR malnourish* OR “malnutrition”/exp OR malnutrition OR stunted OR wasted OR wasting OR “wasting syndrome”/exp OR “wasting syndrome” OR starve* OR starvat* OR “starvation”/exp OR “starvation” OR “cachexia”/exp OR “cachexia” OR “growth faltering”/exp OR “growth faltering” OR marasm* OR “marasmus” OR “kwashiorkor”) AND “controlled study”/de AND ([child]/lim OR [preschool]/lim)
3	(“RUTF”AND “high oleic acid”) OR (“RUTF” AND “low linoleic acid”) OR “RUTF” AND “high oleic peanut” OR “RUTF” AND “palm oil” OR “RUTF” AND “linseed oil” OR “RUTF” AND “flaxseed oil” OR (“RUTF” AND “fish oil capsules”) OR (“RUTF” AND “arachidonic acid”) OR (“RUTF” AND “docosahexaenoic”) acid OR RUTF AND corn/soy blend oil-based) AND (((food* OR feed* OR diet*))) OR ((“ready to use”) AND (therapeutic) AND ((food* OR feed* OR diet*)))
4	*rutf OR rutf OR (plumpy AND nut) OR (ready AND to AND use AND therapeutic AND food) OR (ready AND to AND use AND therapeutic AND feeds) OR chiponde OR nutriset OR proactiva OR edesia OR Peanut butter OR (corn soy blend) OR BP100
5	1 AND 2 AND 3 AND 4

### Data extraction and quality assessment

Four of the review authors (A.N., C.C., B.C.-K., and G.M.) independently extracted the data according to a pre-piloted data-extraction form. All disagreements were addressed by the primary investigator (M.J.L.) and research team for consensus. For each of the relevant studies, the following data were extracted: basic information (authors, contact details, date, and study location), methods, participants’ characteristics, recruitment method, blinding, interventions, outcomes (primary and secondary outcomes), and conclusions. Two review authors (A.N. and M.J.L.) entered the study results into Review Manager 5.4.1 (RevMan 5.4.1), and a third review author (C.C.) checked the data entry for accuracy. If studies reported outcomes at multiple time points, endpoint data were used. The Cochrane Risk-of-Bias 2.0 tool was utilized for the quality assessment of the chosen articles by 5 review authors (B.C.-K., G.M., A.N., C.C., and M.J.L.). The risk of bias was assessed through 5 different domains, including randomization, deviations from the intended interventions, missing outcome data, measurement of outcomes, and the selection of the reported results.^44^ Last, the overall risk of bias for each study was determined based on the criteria determined by Higgins et al.^44^

### Statistical analyses

RevMan 5.4.1 was used for data management and analyses. To summarize data, forest plots were used, and results are reported as risk ratios or odds ratios (ORs) for dichotomous data, and arithmetic means and SDs compared using mean differences were used for continuous data. Results are presented with 95% confidence intervals (CIs). In cases where data could not be pooled, available results are reported in the Results narrative. One clustered randomized trial was included.^45^ While this study was randomized at the cluster level, the authors analyzed the data at an individual level and adequately accounted for clustering in the analysis. In addition, 3 multi-arm studies were included in the review.^43^^,^^46^^,^^47^ All arms in the same meta-analyses were used and compared against the control group, where, for dichotomous outcomes, the control group’s number of events and sample size were divided by 2, and for continuous outcomes the authors divided the control group’s sample size by 2 but the means and SDs remained unchanged. It was attempted to obtain essential missing outcome data from the study authors. Where SDs were not reported, the SDs were calculated from CIs where provided. Heterogeneity between the results of the primary studies was assessed using Cochran’s Q test and quantified with the *I^2^* statistic. Heterogeneity was deemed significant when the *I^2^* statistic value exceeded 50%. A probability value less than 0.1 (*P* < 0.1) was considered to suggest statistically significant heterogeneity. The fixed-effects model was fitted where the *I^2^* statistic value was less than or equal to 50%, while the random‐effects model was used if the *I^2^* statistic value was greater than 50% and if there were sufficient studies to include. Studies conducted in different settings were included, with varying treatment durations as well as different definitions of primary outcome endpoints. This was expected to result in high heterogeneity. Therefore, where data allowed (number of studies > 3) the random-effects models were used to pool estimates from the included studies. Where substantial statistical heterogeneity existed (*I^2^* > 50%), the potential sources of heterogeneity were investigated through subgroup and sensitivity analyses. The treatment intervention effects were compared across the following subgroups: types of fatty acid profiles of RUTFs—profiles with (1) lower n-6:n-3 PUFA ratio, (2) higher n-6:n-3 PUFA ratio, and (3) those additionally with n-3 LCPUFAs. Subgroup analyses were conducted if statistical heterogeneity was detected and the data were sufficient. These were assessed using chi-square tests with a significance level of *P* < 0.10. Sensitivity analyses were explored to assess how adding or omitting certain studies that differ from others affected the overall results by using the leave-one-out method. This was performed according to the influence of studies on heterogeneity, the blinding status of the participants, and the blinding of outcome assessment. All tests were 2-tailed, with a significance level of *P* < 0.05.

## RESULTS

### Results of the search

Figure 1 shows the search flow diagram for this systematic review. Ten reports were included in the review providing a total sample of 10 822 children.^42^^,^^43^^,^^45–52^ The characteristics of the included studies are summarized in Table 3.^42^^,^^43^^,^^45–52^ The studies were all randomized controlled trials with 1 cluster randomized controlled trial^45^ published between 2013 and 2021. Subgroup analyses were conducted according to the fatty acid profile of the alternative RUTFs, which is presented in Table S1 (*see the Supporting Information online*).^42^^,^^43^^,^^45–52^ These subgroups included the RUTFs with (1) lower n-6:n-3 PUFA ratios, such as RUTFs with a lower LA content by adding high-oleic-acid peanuts^42^^,^^46^ and high-oleic-acid soybean oil^51^ or by increasing the ALA content by adding flaxseed^43^^,^^46^^,^^52^ or canola^50^; (2) RUTFs with n-3 LCPUFA supplementation (added and additional encapsulated oil for the child or in the form of a fish paste)^43^^,^^46^^,^^48^; and (3) with higher n-6:n-3 PUFA ratios as a result of the change in ingredients of the standard RUTF recipe.^47^^,^^49^ As comparators, all studies included the standard RUTF formulation adhering to the guidelines provided by the World Health Organization.^53^ Nine studies included a peanut-paste–based RUTF,^42^^,^^43^^,^^45–47^^,^^49–52^ whereas Sigh et al^48^ administered BP-100 (RUTF in the form of a biscuit/bar) as the comparator. The quality of the studies was assessed using the GradePro method (*see*Table S3*in the Supporting Information online*^42^^,^^43^^,^^45–52^). Four studies were excluded with reasons for exclusion (*see*Table S2*in the Supporting Information online*).

**Table 3:** Characteristics of included studies

Reference	Country setting	Study design	Sample size, n	Malnutrition types	Age, mo	Intervention RUTF description	Outcomes
Irena et al^45^	Zambia	Cluster-randomized equivalence trial	Intervention: 824 Comparator: 1103	Severe wasting and edematous malnutrition	6–59	Soy-maize-sorghum-based RUTF	Rate of weight gain, recovery, mortality
Hsiesh et al^42^	Malawi	Randomized, double-blind, clinical effectiveness trial	Intervention: 71 Comparator: 70	Severe wasting and edematous malnutrition	6–59	High-oleic-acid RUTF: high-oleic-acid peanut, palm oil, and linseed oil product (Nutriset, France)	Fatty acid status, rate of weight, length/height, and MUAC gain, *z* scores, recovery, mortality
Jones et al^43^	Kenya	Three-armed randomized double-blind controlled trial	Intervention 1: 20 Intervention 2: 20 Comparator: 20	Severe wasting and edematous malnutrition	6–59	Intervention 1: flaxseed oil containing RUTF (F-RUTF): standard formulation (project Valid Nutrition, Lilongwe, Malawi) with cold-pressed flaxseed oil (Seed Oil SA, Somerset West, South Africa) Intervention 2: standard formulation produced (project Valid Nutrition, Malawi) with cold-pressed flaxseed oil (Seed Oil SA, South Africa), with 2 0.5-mL fish-oil capsules (Seven Seas, UK) (214 mg EPA plus DHA at a ratio of 1.7:1 with 4 IU vitamin E)	Erythrocyte PUFA composition, rate of MUAC gain, *z* scores, recovery, diarrhea, vomiting, LRTI, URTI, rash, inflammatory markers, mortality, safety, adverse events, acceptability
Bahwere et al^52^	Democratic Republic of the Congo	Parallel-group simple randomized controlled trial	Intervention: 445 Comparison: 441	Severe wasting and edematous malnutrition	6–59	Soya-maize-sorghum RUTF	Recovery, fever, diarrhea, mortality, acceptability
Bahwere et al^47^	Malawi	Parallel-group, simple randomized controlled trial	Intervention 1: 458 Intervention 2: 435 Comparator: 454	Severe wasting and edematous malnutrition	6–59	Intervention 1: milk-free soya, maize, and sorghum RUTF (FSMS-RUTF) Intervention 2: milk, soya, maize, and sorghum RUTF (MSMS-RUTF)	Recovery, fever, diarrhea, cough, mortality, acceptability
Sigh et al^48^	Cambodia	Randomized, single-blind home-based trial	Intervention: 60 Comparator: 61	Severe wasting only	6–59	NumTrey: locally produced fish-based RUTF containing rice, soybean, mung beans, canola oil, and dried, powered small indigenous fish	Rate of weight, length/height, and MUAC gain, *z* scores, acceptability
Kohlmann et al^50^	Ghana	Randomized, double-blind controlled trial	Intervention: 199 Comparator: 202	Severe wasting and edematous malnutrition	6–59	Half of the peanuts were replaced with locally available soybean and sorghum flour, with whey protein concentrate and nonfat dried milk. Also included canola oil, sugar, vitamin and mineral premix, and a nonnutritive emulsifier.	Rate of weight and MUAC gain, recovery, mortality
Hendrixon et al^51^	Sierra Leone	Randomized, triple-blinded, controlled clinical noninferiority trial	Intervention: 721 Comparator: 685	Severe wasting and edematous malnutrition	6–59	Oats, peanuts, sugar, milk powder, vegetable oil, a premix containing concentrated minerals and vitamins, and emulsifiers RUTF	Rate of weight, length/height, and MUAC gain, recovery, mortality, fever, diarrhea, vomiting, cough, acceptability
Oakley et al^49^	Malawi	Randomized, double-blind, controlled, clinical, quasi-effectiveness trial	Intervention: 929 Comparator: 945	Severe wasting and edematous malnutrition	6–59	10% Milk RUTF	Rate of weight, length/height, and MUAC gain, *z* scores, recovery, mortality, cough, acceptability
Stephenson et al^46^	Malawi	Triple-blind randomized controlled trial	Intervention 1: 809 Intervention 2: 860 Comparator: 896	Severe wasting and edematous malnutrition	6–59	Intervention 1 (DHA-HO-RUTF): DHA in similar amounts as in breast milk by the addition of encapsulated oil Intervention 2 (HO-RUTF): replaced regular peanuts with high oleic peanuts, the addition of perilla oil and exclusion of canola oil	Fatty acid status, global development, gross motor development, motor development, language development, social development, cognition, rate of weight, length/height, and MUAC gain, recovery, mortality, fever, diarrhea, cough

*Abbreviations:* DHA, docosahexaenoic acid; EPA, eicosapentaenoic acid; LRTI, lower respiratory tract infection; MUAC, midupper arm circumference; PUFA, polyunsaturated fatty acid; RUTF, ready-to-use therapeutic food; URTI, upper respiratory tract infection.

### Risk of bias

**Figure 2:**
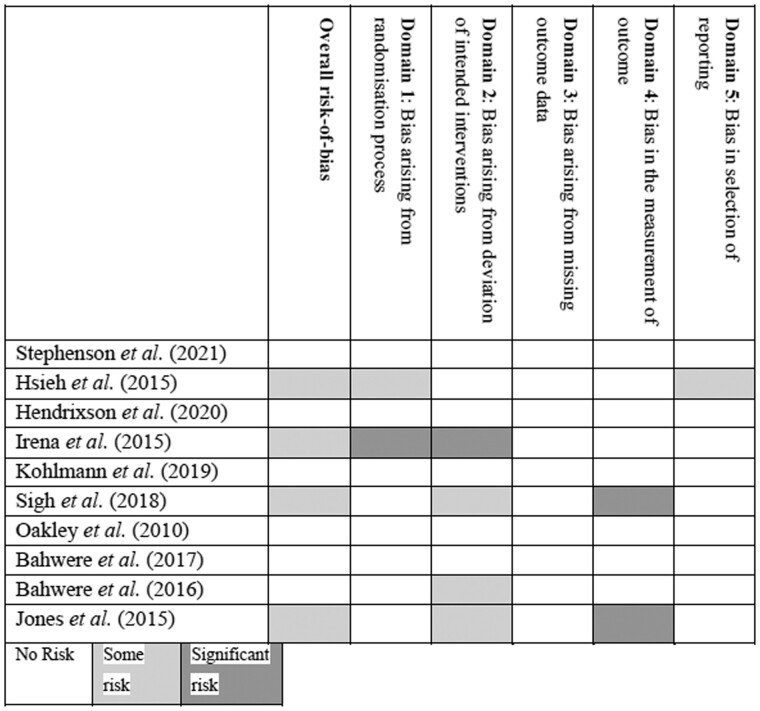
Risk-of-bias assessment of the included studies.

Figure 2 ^42^ ^,^ ^43^ ^,^ ^45–52^ shows the risk of bias in the included studies according to the 5 domains as stipulated in the RoB2 tool.^44^ When all of the studies were evaluated for the overall risk of bias, the studies by Hsieh et al,^42^ Irena et al,^45^ Sigh et al,^48^ and Jones et al^43^ had some concerns for bias.

### Effects of the interventions

#### Fatty acid status

Three studies^42^^,^^43^^,^^46^ reported the plasma or erythrocyte phospholipid fatty acid composition in participants. Stephenson et al^46^ and Hsieh et al^42^ reported plasma fatty acid status, and their data were pooled. However, Jones et al^43^ reported erythrocyte fatty acid status as medians (interquartile range), which could not be pooled. In this study, the groups receiving FFO-RUTF (flaxseed RUTF with fish oil) and F-RUTF (flaxseed RUTF) had a higher erythrocyte DHA content compared with those children receiving the standard formulation (6.3 [6.02–7.33] and 4.51 [3.92–4.85] vs 3.88 [2.36–5.70]; *P* ≤ 0.001), while only the FFO-RUTF group had a higher plasma DHA content at the end of 84 days compared with baseline content (*P* ≤ 0.001).^43^

In the meta-analysis, there was a higher plasma LA content in children receiving the standard RUTF compared with children receiving alternative RUTF formulations (overall mean difference: –0.83 [–1.48, –0.18]; *P* = 0.01). When stratified by fatty acid profiles, the overall mean difference in studies administering an alternative RUTF with n-3 LCPUFA compared with the standard RUTF was –0.95 (–1.91, 0.01; *P* = 0.05). There was a higher plasma ALA content in children consuming alternative formulations of RUTF compared with standard RUTF, with an overall mean difference of 0.23 (0.18, 0.28; *P* < 0.0001). There were no subgroup differences by fatty acid profile with ALA content (test for subgroup differences: χ^2^ = 0.44, *P* = 0.51, *I^2^* = 0%).

Plasma arachidonic acid content was higher in children receiving the standard RUTF compared with alternative RUTF formulations, but high heterogeneity was observed (overall mean difference: –0.73 [–1.22, –0.24]; *P* = 0.004). There was a higher plasma arachidonic acid content in children receiving the standard RUTF compared with children receiving the lower n-6:n-3 PUFA ratio RUTFs, but this was not observed for children in studies receiving alternative RUTFs with n-3 LCPUFAs. A test for subgroup differences showed a significant mean difference between the 2 subgroups (*P* = 0.01), but the heterogeneity was high (*I^2^* = 83%).

**Figure 3:**
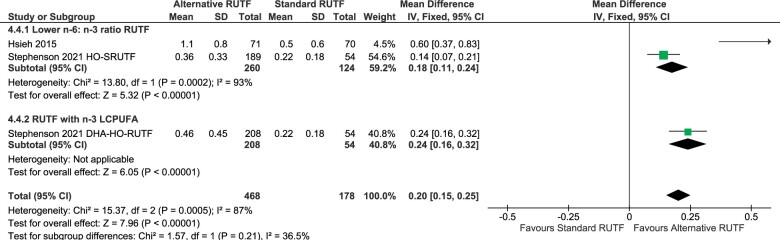
**Meta-analysis of plasma phospholipid eicosapentaenoic acid content in the percentage of total fatty acids.** DHA-HO-RUTF: high-oleic-acid RUTF; Lower n-6:n-3 ratio RUTF, added HO peanuts, perilla oil, linseed oil, combination soy, maize, sorghum, flaxseed oil, canola oil, HO soybeans; RUTF with n-3 LCPUFA, added fish-oil capsules, fish paste from dried fish. *Abbreviations:* CI, confidence interval; DHA, docosahexaenoic acid; HO, high-oleic-acid; IV, inverse variance; LCPUFA, long-chain polyunsaturated fatty acid; RUTF, ready-to-eat food; SD, standard deviation

Plasma phospholipid EPA content was higher in children receiving alternative RUTF formulations compared with the standard RUTF (overall mean difference: 0.20 [0.15, 0.25]; *P* < 0.00001) (Fig. 3^42^^,^^46^). The plasma phospholipid EPA content did not differ significantly between children receiving RUTFs with a lower n-6:n-3 PUFA ratio and RUTFs with n-3 LCPUFAs. Both subgroups, however, showed a higher plasma EPA content in children receiving alternative RUTF formulations compared with the standard RUTF.

**Figure 4:**
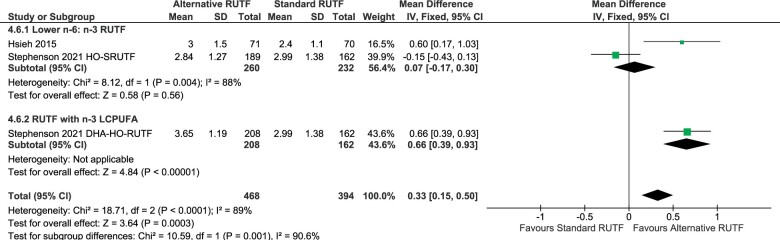
**Meta-analysis of plasma phospholipid DHA content in the percentage of total fatty acids.** DHA-HO-RUTF: high-oleic-acid RUTF; Lower n-6:n-3 ratio RUTF, added HO peanuts, perilla oil, linseed oil, combination soy, maize, sorghum, flaxseed oil, canola oil, HO soybeans; Higher n-6:n-3 ratio RUTF, added soy; RUTF with n-3 LCPUFA, added fish-oil capsules, fish paste from dried fish. *Abbreviations:* CI, confidence interval; DHA, docosahexaenoic acid; HO, high-oleic-acid; IV, inverse variance; LCPUFA, long-chain polyunsaturated fatty acid; RUTF, ready-to-eat food; SD, standard deviation

Both Stephenson et al^46^ and Hsieh et al^42^ reported higher plasma DHA content in children receiving alternative formulations as compared with standard RUTF, but the heterogeneity was high (overall mean difference: 0.33 [0.15, 0.50]; *P* = 0.0003) (Fig. 4^42^^,^^46^). Significant subgroup differences in plasma phospholipid DHA content were observed between children receiving lower n-6:n-3 PUFA ratio RUTF and RUTF with n-3 LCPUFA but the heterogeneity was very high (test of subgroup differences: *P* = 0.001, *I^2^* = 90%) (Fig. 4).

#### Neurodevelopmental outcomes

Only 1 multi-arm study^46^ assessed neurodevelopment as a primary outcome using the Malawi Developmental Assessment Tool (MDAT) global development and problem-solving assessment scores, including fine motor, gross motor, social, and language development. Therefore, results are presented here narratively. The study compared the standard RUTF with 2 alternative RUTF formulations—that is, DHA-HO-RUTF (high-oleic-acid formulation with added fish oil) and HO-RUTF (high-oleic-acid formulation). There were significantly higher scores of the global assessment and the gross motor domains in children receiving DHA-HO-RUTF 6 months post–SAM outcome compared with the standard formulation (mean difference: 0.19 [0.0, 0.38] vs 0.29 [0.03 to 0.55]), but no differences were observed in these domains between children receiving HO-RUTF and the standard formulation (0.08 [–0.11, 0.27] vs 0.02 [–0.24 to 0.29]). The children in both of the intervention arms had significantly higher scores in the social domain compared with the standard formulation (0.16 [0.00 to 0.31] for DHA-HO-RUTF and 0.24 [0.09 to 0.40] for HO-RUTF). No differences were observed between children receiving the intervention and the standard formulations for the fine motor and language domain assessments. Similarly, no associations were observed with problem-solving assessment intention scores or the eye-tracking outcomes for children receiving the intervention in comparison with the standard formulation. However, the mean infant-oriented attention response time was slightly higher in the children in the intervention arms compared with those in the standard formulation arm.

#### Nutritional recovery and growth

Growth outcomes reported by Jones et al^43^ could not be included in the meta-analysis due to differences in reporting. However, no significant differences between the F-RUTF, FFO-RUTF, or standard RUTF groups regarding the change in anthropometrical status were reported.^43^

#### Rate of weight gain

**Figure 5:**
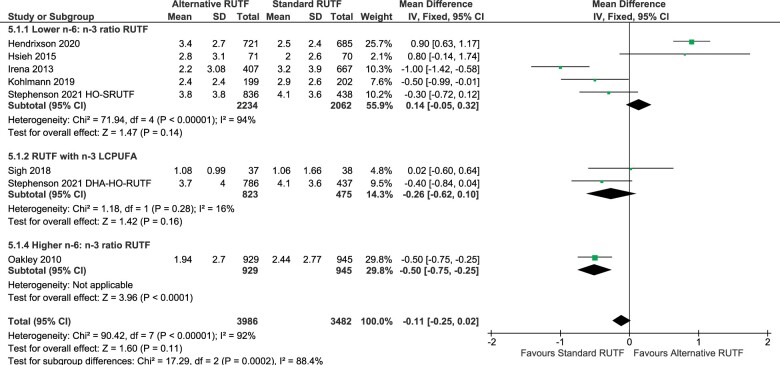
**Meta-analysis of the rate of weight gain in grams per kilogram body weight per day.** DHA-HO-RUTF: high-oleic-acid RUTF; Lower n-6:n-3 ratio RUTF, added HO peanuts, perilla oil, linseed oil, combination soy, maize, sorghum, flaxseed oil, canola oil, HO soybeans; RUTF with n-3 LCPUFA, added fish-oil capsules, fish paste from dried fish. *Abbreviations:* CI, confidence interval; DHA, docosahexaenoic acid; HO, high-oleic-acid; IV, inverse variance; LCPUFA, long-chain polyunsaturated fatty acid; RUTF, ready-to-eat food; SD, standard deviation

The rate of weight gain in grams per kilogram of body weight per day (g/kg/day) was assessed in 7 studies (including a multi-arm study), which were included in the meta-analysis (Fig. 5).^42^^,^^45^^,^^46^^,^^48–51^ Three of these studies reported significantly lower weight gain in children receiving alternative RUTF formulations compared with standard RUTF, Kohlmann et al^50^ (–0.50 g/kg/day [–0.99, –0.01]), Oakley et al^49^ (–0.50 g/kg/day [–0.75, –0.25]), and Irena et al^45^ (–1.00 g/kg/day [–1.42, –0.58]). The overall pooled estimate results from a random-effects model showed that the overall rate of weight gain was, however, not significantly different between the interventions and the standard RUTF (overall mean difference: –0.15 g/kg/day [–0.67, 0.37]; *P* = 0.57). As part of sensitivity analyses the study by Hendrixson et al^51^ was excluded, which influenced the overall effect (heterogeneity reduction from 92% to 63%). The pooled estimates showed a significant mean difference in the rate of weight gain, with higher weight gain in children receiving standard RUTF compared with the alternative RUTF interventions (overall mean difference: –0.39 g/kg/day; 95% CI: –0.67, –0.10; *P* = 0.007). However, the subgroup effect remained nonsignificant (test for subgroup differences: *P* = 0.56). Excluding 1 study at a time according to the blinding status of the participants and blinding of outcome assessment did not affect the conclusion of the subgroup effect by fatty acid profiles. Bahwere et al^47^^,^^52^ reported the rate of weight gain separately for age groups; therefore, these data could not be pooled. Bahwere et al^47^ found that weight-gain rates in the milk-free soya, maize, and sorghum RUTF (FSMS-RUTF) trial products appeared to be lower than those seen in the children receiving Peanut and milk based RUTF (PM-RUTF).

#### Rate of height gain

Three studies (including 1 multi-arm study) measured and reported the rate of length/height gain per day (mm/day).^42^^,^^46^^,^^49^ A random-effects meta-analysis of the 3 studies did not show a significant difference in the rate of length/height gain between the alternative and the standard RUTF (overall mean difference: 0.01 mm/day [–0.04 to 0.05]; *P* = 0.85). A subgroup analysis by fatty acid profiles of the RUTF showed a significant subgroup effect difference (test for subgroup differences: *P* = 0.01) between studies with alternative RUTFs with a lower n-6:n-3 PUFA ratio (overall mean difference: 0.03 mm/day [–0.04, 0.10]; *P* = 0.38) versus the study with children receiving n-3 LCPUFA as the alternative RUTF formulation^46^ (mean difference: 0.02 mm/day [–0.02, 0.06]) and the study with a higher n-6:n-3 PUFA ratio as the alternative RUTF (mean difference: –0.04 mm/day [–0.06, –0.02]).^49^ There was an insufficient number of studies included in the higher n-6:n-3 PUFA ratio and alternative RUTF with n-3 LCPUFA subgroups; therefore, the test of subgroup effects may be concerning for these subgroups. Bahwere et al^52^ was not included in the meta-analysis as they reported a rate of linear growth per age group. In this study, there was no significant difference in change in the rate of length gain between groups in any of the age groups (soya-maize-sorghum-based RUTF [SMS-RUTF] and standard peanut paste-based RUTF [P-RUTF]).^52^

#### Rate of MUAC gain

Seven studies reported MUAC gain, and the evidence was mixed (*see*Fig. S1*in the Supporting Information online*).^42^^,^^46^^,^^48–51^ Two studies^49^^,^^50^ reported significant mean differences in MUAC gain between children who consumed standard RUTF and alternative RUTF interventions. Overall, the pooled estimate did not show a significant difference, but high heterogeneity was observed, highlighting the uncertainty over the estimate from the random-effects model (overall mean difference: –0.01 mm/day [–0.05, 0.02]; *P* < 0.51).

#### Weight-for-height z scores

Four studies were included for WHZ outcomes,^42^^,^^48^^,^^49^^,^^52^ 2 of which reported significant differences in mean WHZ in favor of the alternative RUTF formulations (*see*Fig. S2*in the Supporting Information online*).^42^^,^^48^ The random-effects pooled estimate showed a borderline significant overall mean difference in favor of the alternative RUTF (0.12 [–0.00, 0.25]; *P* = 0.06). In a subgroup analysis, studies of RUTFs with a lower n-6:n-3 PUFA ratio showed a significant mean difference in WHZ between the children receiving the alternative formulation and the standard formulation (0.40 [0.11, 0.69]; *P* = 0.007). An overall test for subgroup differences for the different fatty acid profiles showed a difference in WHZ between children in the lower n-6:n-3 PUFA ratio RUTF subgroup^42^^,^^52^ versus children in the higher n-6:n-3 PUFA ratio RUTF subgroup^49^ and the RUTF with n-3 LCPUFA subgroup^48^ (*P* = 0.010), indicating that fatty acid profile may modify the effect of formulation on the child’s WHZ. However, the sample size was limited for the 2 subgroups of higher n-6:n-3 PUFA ratio and RUTF with n-3 LCPUFA; therefore, the subgroup effect needs to be interpreted cautiously.

#### Height-for-age z score and weight-for-age z score

The height-for-age *z* score (HAZ) was assessed in 2 studies.^49^^,^^52^ Neither the included studies nor the overall fixed-effects model showed a significant difference in HAZ (overall mean difference: –0.11 [–0.25, 0.02]; *P* = 0.09). In a subgroup analysis by fatty acid profiles, there was also no significant difference (*P* = 0.31). Two studies reported weight-for-age *z* score (WAZ).^49^^,^^52^ Both studies reported no differences in WAZ, and the fixed-effects model overall effect was also nonsignificant (–0.10 [–0.20, 0.01]; *P* = 0.07). In a subgroup analysis by fatty acid profile, there was also no significant difference (*P* = 0.80).

#### Recovery

Eight studies (including 2 multi-arm studies) that assessed recovery were included in this review (*see*Fig. S3*in the Supporting Information online*).^42^^,^^45–47^^,^^49–52^ One study reported higher odds of recovery in favor of the children receiving alternative RUTF compared with the standard RUTF (odds ratio [95% CI]: 1.53 [1.24, 1.89]),^51^ whereas 2 studies reported significantly higher odds of recovery in the children receiving standard RUTF compared with the alternative RUTF.^45^^,^^50^ Random-effects model pooled estimates showed a borderline significant difference in odds of recovery between the children receiving the standard RUTF and alternative RUTF intervention arms (0.91 [0.83, 0.99]), but the heterogeneity was high (χ^2^ = 38.68, *P* < 0.001, *I^2^* = 77%). A subgroup analysis by fatty acid profile did not show significant differences (*P* = 0.90). Bahwere et al^47^ reported differences in estimated marginal means and their clustered robust adjusted 95% CIs in recovery rates stratified by age group. There were no significant differences in recovery rates observed in this study.^47^

#### Mortality

Nine studies (including 3 multi-arm studies) reported mortality outcomes (*see*Fig. S4*in the Supporting Information online*),^42^^,^^43^^,^^45–47^^,^^49–52^ of which 1 study reported a significant difference with higher mortality reported in children receiving standard RUTF.^45^ None of the other studies reported any differences in mortality. However, in the fixed-effects model, a statistically significant difference in mortality with a higher risk of mortality in children receiving standard RUTF compared with the alternative RUTF formulations was observed (0.79 [0.67, 0.94]; *P* = 0.008). Stratifying the analyses separately by fatty acid profile revealed borderline subgroup differences in mortality between alternative formulation interventions and the standard intervention group (*P* = 0.10). The intervention effect on mortality favored children receiving the alternative formulations over standard RUTF and this effect was greater for children receiving formulations with lower n-6:n-3 PUFA ratios. There were no significant associations for the remaining fatty acid profile subgroups.

#### Comorbidities

The studies included in this review reported comorbidities of fever, diarrhea, lower respiratory tract infections, upper respiratory tract infections (URTIs), rash, and cough. Jones et al^43^ was the only study that reported URTIs, lower respiratory tract infections, and rash outcomes, and no associations with any of the interventions were reported. However, in the fixed-effects model, a statistically significant difference in URTIs with a higher risk of URTIs in children receiving standard RUTF compared with the alternative RUTF formulations was observed (2.5 [1.36, 4.58]; *P* = 0.003) (*see*Fig. S5*in the Supporting Information online*^43^). There were no other significant associations between comorbidities and RUTFs in the total group or subgroup analyses. Bahwere et al^52^ reported morbidity data per age group and was not included in the meta-analysis. There was no significant difference in the occurrence and duration of diarrhea and fever between groups (SMS-RUTF and P-RUTF) in this study.^52^

#### Adverse events

Vomiting and anaphylaxis outcomes were assessed in 2 of the included studies.^43^^,^^51^ No signs of anaphylaxis were reported by Hendrixson et al.^51^ In the fixed-effects model, no association with vomiting was found (overall effect: 1.20 [0.87, 1.66]; *P* = 0.26). There were no differences between subgroups for adverse event outcomes (*P* = 0.22).

#### Acceptability

Six studies recorded the acceptability of the alternative RUTFs with altered fatty acid profiles.^42^^,^^47–49^^,^^51^^,^^52^ Four of these studies did not find any differences in acceptability between alternative RUTFs and the standard RUTF comparator.^42^^,^^47^^,^^49^^,^^51^ However, Sigh et al^48^ showed that the proportion of children expressing that they liked the altered fatty acid RUTF (with fish paste) in their study increased after 8 weeks of the intervention. Additionally, notable acceptability and tolerance differences were observed in a study in Malawi,^52^ where fewer children younger than 24 months experienced flatulence on the altered RUTF (soya, maize, and sorghum-based). Among those who defaulted in this study, dislike of RUTF was reported to be higher for the alternative RUTF (19.4% vs 13.3% for the peanut RUTF).^52^ Stephenson et al^46^ reported that their alternative RUTF (high-oleic-acid) was already found to be acceptable previously^42^ and, therefore, they did not include an additional acceptability study.

## DISCUSSION

This systematic review and meta-analysis summarized the findings of 10 studies concerning the effects of alternative RUTF formulations with different EFA profiles on the outcomes of 10 822 children (6 to 59 months) diagnosed with severe wasting. Studies were grouped in subgroups according to the fatty acid profiles of the alternative RUTFs—namely, RUTFs with (1) lower and (2) higher n-6:n-3 PUFA ratios compared with the standard RUTF and RUTFs with additional preformed n-3 LCPUFA (as fish oil/paste). The main finding was that the plasma phospholipid DHA content in children was most likely to increase only when fish oil was administered additionally to the RUTF, while lowering the LA content and/or increasing the ALA content may only result in significant changes in LA, ALA, and EPA status. Furthermore, lowering the n-6:n-3 PUFA ratio of RUTFs may contribute to improved social development MDAT scores and WHZ and lower mortality rates of children with wasting. The addition of preformed n-3 LCPUFAs in the form of fish oil or fish paste to RUTFs may benefit more neurodevelopmental outcomes and improve WHZ.

Three studies reported fatty acid status^42^^,^^43^^,^^46^; however, the data from Jones et al^43^ could not be pooled. From the meta-analysis, significantly higher plasma phospholipid EPA content was found in the children receiving alternative RUTF formulations with lower n-6:n-3 PUFA ratios and in the group receiving RUTFs with added n-3 LCPUFAs. It is well known that PUFA biosynthesis is influenced by various factors, including diseases, diet, and nutritional status.^54^ As poor nutritional status (nutrient deficiencies such as zinc, magnesium, and protein among others), together with a high probability of infections in the studied population exist, one may suspect lower desaturase activity in malnourished children. However, the findings of this systematic review are in line with previous research indicating that children with SAM have healthy desaturase activity; therefore, when providing RUTFs with improved n-6:n-3 PUFA ratios, EPA synthesis from n-3 PUFA was favored, resulting in improved EPA status.^42^^,^^54^ The DHA status was, however, only significantly higher in the subgroup receiving RUTFs with the preformed n-3 LCPUFA. This is in contrast to the results by Jones et al^43^ who reported significantly higher DHA content in erythrocytes in both groups receiving RUTF with flaxseed oil (FO-RUTF) and in those receiving RUTF with added fish oil (FFO-RUTF) compared with the standard RUTF group. However, in this study, the FFO-RUTF group was the only group that had a significantly higher erythrocyte DHA content after 84 days of treatment when compared with baseline, while this was not the case in the flaxseed oil arm (FO-RUTF).^43^ Due to the importance of DHA for neurodevelopment, the already low DHA status of malnourished children, and that standard RUTF may even further worsen DHA status, this is a significant finding to consider in future RUTF formulations.^19^^,^^21^^,^^42^ It is important to note that the heterogeneity for arachidonic acid, EPA, and DHA outcomes was high and that the number of studies included for these comparisons may have been insufficient to draw definite conclusions. Therefore, the quality of evidence was rated low or moderate for most fatty acid status outcomes. The underlying molecular aspects of these findings are not the focus of this review, but potential genetic variations in the *FADS* gene cluster could affect desaturase activity and influence the EFA intake to LCPUFA conversion, which contributes to DHA status. The antagonistic molecular and genetic effects of LA on n-3 PUFAs are well known.

Only 1 study reported neurodevelopmental outcomes.^46^ In this study, 2565 children diagnosed with SAM were enrolled in 1 of 3 arms: (1) an intervention arm using high-oleic-acid peanuts with added perilla oil (lower LA, higher ALA content, and a lower n-6:n-3 PUFA ratio compared with standard RUTF [HO-RUTF]), (2) an intervention arm including the HO-RUTF with additional fish oil (with n-3 LCPUFA [DHA-HO-RUTF]), and (3) the comparator, which was the standard RUTF. The authors reported that the children in the DHA-HO-RUTF arm had higher global and gross motor MDAT scores and both DHA-HO-RUTF and HO-RUTF arms had higher social MDAT scores compared with the children receiving the standard RUTF.^46^ The findings of these neurodevelopmental outcomes should be cautiously interpreted as they are from 1 research study, which lowers the generalizability of the results to other populations. Nevertheless, these results are explained by the fatty acid status results as DHA is needed to support neurodevelopment and an improved DHA status will, therefore, benefit neurodevelopmental outcomes.^19^ As children with SAM have been reported to present with impaired short- and longer-term neurodevelopment, these results are promising for future RUTF formulations.^27^^,^^30^

As nutritional recovery is considered the main outcome in children with severe wasting, the rate of weight, length/height, and MUAC gain, *z* scores, and number of children who recovered were included as outcomes. No significant effects on the rate of weight gain and the rate of length/height gain overall or between subgroups were found. However, in the 1 study included in the higher n-6:n-3 PUFA RUTF subgroup, the weight, length/height, and MUAC gain rates were significantly higher in the standard RUTF group than in the alternative RUTF group.^49^ It should be kept in mind that the aim of the alternative RUTF used in this single study was not to alter the fatty acid profile and, therefore, these differences may be due to alterations of other ingredients and nutrients in this formulation. No significant effect on recovery overall or between subgroups was found. With regard to the WHZ, the children in both subgroups receiving RUTFs with a lower n-6:n-3 PUFA ratio and RUTF with n-3 LCPUFA had a higher WHZ compared with those receiving the standard RUTF. These results were only pooled from 2 studies and were graded as moderate certainty of the evidence but may still be promising. The mortality rate was also found to be significantly lower in children receiving RUTFs with a lower n-6:n-3 PUFA ratio. Even though the mechanism underpinning the better WHZ and lowering effect on mortality is unknown, this further demonstrates the possible benefit of lowering the n-6:n-3 PUFA ratio in RUTFs.

Outcomes regarding immunity and adverse events were also included in this meta-analysis due to the well-known role of fatty acid intake and status in immunity.^15^ There were no significant effects on fever, rash, cough, lower respiratory tract infections, vomiting, and diarrhea. URTIs were significantly lower in the children receiving alternative RUTFs with a lower n-6:n-3 PUFA ratio or with n-3 LCPUFAs. This may show the beneficial effects of n-3 LCPUFA on the immune system and corresponds to fatty acid status findings. However, these results should be interpreted with caution. The quality of evidence for this outcome was downgraded to low as the results came from only 1 study and the sample size was small, with wide CIs (imprecision).

### Quality of evidence

Most of the included studies were considered a low risk of bias, except for Hsieh et al,^42^ Irena et al,^42^ Sigh et al,^48^ and Jones et al,^43^ which might cause some concerns for bias. Furthermore, making use of the GradePro tool, it was found that the quality of the evidence for those outcomes reported in the “Summary of findings” tables was mixed, ranging from low- to high-quality findings.

## CONCLUSION

**Figure 6:**
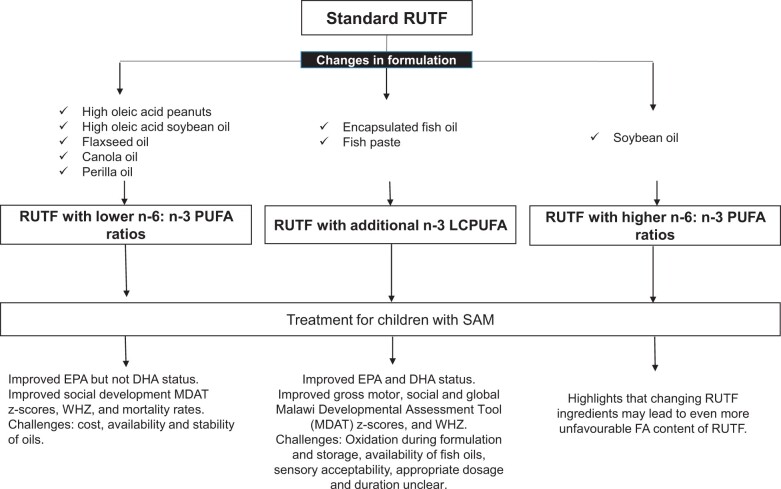
**Summary of the findings of systematic review.** *Abbreviations:* DHA, docosahexaenoic acid; EPA, eicosapentaenoic acid; FA, fatty acid; LCPUFA, long-chain polyunsaturated fatty acid; MDAT, Malawi Developmental Assessment Tool; PUFA, polyunsaturated fatty acid; RUTF, ready-to-use-therapeutic food; SAM, severe acute malnutrition; WHZ, weight-for-height *z* score.

In summary (Fig. 6), RUTFs with a lower n-6:n-3 PUFA ratio did not result in higher plasma phospholipid DHA status. Even though the quality of evidence was not always high, other benefits of lowering the n-6:n-3 PUFA ratio, such as improved social development MDAT scores and WHZ and lower URTIs and mortality rates, were found. When interpreting these results, it is imperative to consider whether the lower n-6:n-3 PUFA ratios were driven by a lower LA content (eg, high-oleic-acid peanuts/oil formulations) or by a higher ALA (formulations with flaxseed and perilla oils) or both. Even though high-oleic-acid peanuts/oils have higher stability and a longer shelf-life, which are suitable for RUTFs, the availability and cost should be considered.^42^^,^^46^ Furthermore, the cost, availability, and stability of higher n-3 PUFA oils such as flaxseed oil (which is prone to increase in peroxide levels over time) may be a challenge.^43^ The second option to alter the fatty acid profile of RUTFs is to add the preformed n-3 LCPUFAs (as fish oil or paste). Higher plasma phospholipid DHA content was evident in this subgroup that also presented with better gross motor, social, and global MDAT scores, higher WHZ, and lower rates of URTIs (low-quality evidence). More research should focus on supporting these findings. Adding fish oil to products may bring about challenges with oxidation during formulation and storage, which can be addressed by encapsulation to protect against oxidation.^46^ However, sourcing capsules may have cost and feasibility implications, and considering the short period of supplementation and the already low n-3 LCPUFA status of the malnourished children, the significance of such interventions may be questionable. Another practical implication is the availability of fish oils. Nevertheless, Sigh et al^48^ included a fish paste from powdered indigenous fish (NumTrey), which may be a more feasible and available option to consider.^55^ More studies are needed as a possible alternative to adding encapsulated fish oil. With the addition of fish oils/pastes to RUTF, further thought should be given to the sensory acceptability of such products and methods, such as masking the fish taste with other flavors.^56^ Further investigation into methods to increase the stability of RUTFs with added fish oil/paste during formulation and storage is required. Last, alternative RUTFs with higher n-6:n-3 PUFA ratios were not mainly formulated to change the RUTF fatty acid profile and, therefore, it was difficult to conclude as to whether differences in this subgroup were driven by changes in fatty acid profiles or other nutrients and ingredients. Nevertheless, this highlights that changing the ingredients in alternative RUTFs may lead to even more unfavorable fatty acid profiles. Given the already low n-3 PUFA status of malnourished children, these formulations may further amplify the problem, and therefore, fatty acid profiles should be considered in future designs of alternative RUTFs, irrespective of the aim of the altered formulation. The clinical implications of altering the fatty acid profile of RUTFs to lower LA and favor n-3 PUFA content may benefit long-term cognitive development, infection risk, and growth in children treated for SAM and, therefore, warrants further investigation and consideration. Considering the findings of currently available studies, it can be recommended that the LA content should be reduced and preformed DHA included in RUTFs. Further research is required to establish the appropriate concentrations and length of administration required for optimal outcomes and to address practical implementation.

## Supplementary Material

nuad151_Supplementary_Data

## Supporting Information

The following Supporting Information is available through the online version of this article at the publisher’s website.

*Table S1* **Fatty acid content of the ready-to-use therapeutic foods used in the included studies**

*Table S2* **Excluded studies with reasons**

*Table S3* **Summary of findings table**

*Figure S1* **Meta-analysis of the rate of midupper arm circumference gain in millimeters per day**

*Figure S2* **Meta-analysis of weight-for-height *z* scores**

*Figure S3* **Meta-analysis of recovery**

*Figure S4* **Meta-analysis of the mortality rate**

*Figure S5* **Meta-analysis of upper respiratory tract infections**
