# Supplementary material for: Effect of fatty acid profiles in varying recipes of ready-to-use therapeutic foods on neurodevelopmental and clinical outcomes of children (6–59 months) with severe wasting: a systematic review
Source: Nutr Rev. 2023 Dec 22;82(12):1784–99. doi: 10.1093/nutrit/nuad151 (PMC11551450; doi:10.1093/nutrit/nuad151)
Supplement: nuad151_Supplementary_Data [file nuad151_supplementary_data.zip › nuad151_Supplementary_Data/Table S1.docx]

**Table S1****: Fatty acid content of the ready-to-use therapeutic foods used in the included studies**

|  | **Oakley *et al.*^S1^** | | **Hsieh *et al.*^S2^** | | **Irena e*t al.***^S3^ | | **Jones *et al.***^S4^ | | | | **Bahwere *et al.*^S5^** | |
| --- | --- | --- | --- | --- | --- | --- | --- | --- | --- | --- | --- | --- |
|  | **I** | **C** | **I** | **C** | **I** | **C** | **I** | **I** | | **C** | **I** | **C** |
|  | 10% milk RUTF | 25% milk RUTF | HO-RUTF | S-RUTF | SMS-RUTF | P-RUTF^c^ | F-RUTF | FFO-RUTF | | S-RUTF | SMS-RUTF | P-RUTF^c^ |
| Total energy (kcal/ 100g) | 2000 kJ | 2000 kJ |  |  | 521 | 530 |  |  | |  | 553 | 530 |
| Total fat (g/100g) | 40 | 40 |  |  | 57.0 | 56.0 |  |  | |  |  |  |
| Fat % of total energy (%) |  |  |  |  |  |  |  |  | |  | 59.1 | 56.0 |
| SFA (g/100g) |  |  | 10.1 | 15.7 |  |  |  |  | |  |  |  |
| MUFA (g/100g) |  |  | 15.8 | 17.4 |  |  |  |  | |  |  |  |
| PUFA (g/100g) |  |  |  |  |  |  |  |  | |  |  |  |
| Total n-6 PUFA (g/100g) |  |  | 4.4 |  |  |  |  |  | |  |  |  |
| n-6 % of total energy (%) |  |  |  |  | 10.4 |  | 8.2 | 7.9 | | 8.2 | 12.3 |  |
| Linoleic acid |  |  | 4.4 | 8.9 |  |  | 14.0 | 14.4 | | 14.9 |  |  |
| Arachidonic acid |  |  |  |  |  |  | 0.8 | 0.9 | | 0.5 |  |  |
| Total n-3 PUFA (g/100g) |  |  |  |  |  |  |  |  | |  |  |  |
| n-3 % of total energy (%) |  |  |  |  | 1.1 |  | 3.3 | 3.9 | | 0.7 | 3.1 |  |
| Alpha-linolenic acid |  |  | 4.4 | 0.2 |  |  | 6.2 | 6.1 | | 1.3 |  |  |
| DHA (mg/100g) |  |  |  |  |  |  | 0 | 0.4 | | 0 |  |  |
| EPA (mg/100g) |  |  |  |  |  |  | 0 | 0.7 | | 0 |  |  |
| **n-6:n-3 ratio** |  |  | 1:1^b^ | 53:1^b^ | 9.6:1 |  | 2.45:1 | 2.02:1 | | 11.72:1 | 4.0:1 |  |
|  |  |  |  |  |  |  |  |  | |  |  |  |
| **Sub-group** | **Higher** n-6: n-3 ratio | | **Lower** n-6: n-3 ratio | | **Lower** n-6: n-3 ratio | | **Lower** n-6: n-3 ratio | | **with n-3 LCPUFA** | | **Lower** n-6:n-3 ratio | |
| **Rational for categorisation** | *Due to the added soybeans of alternative RUTF* | | *Due to high oleic acid peanuts and linseed oil to alternative RUTF.* | | *Lower n-6:n:3 ratio when compared with the average n-6:n-3 ratio of standard RUTF* | | *Due to added flaxseed oil* | | *Due to the flaxseed oil and fish oil capsule* | | *Due to added linseed in the alternative RUTF* | |

## Fatty acid content of the ready-to-use therapeutic foods used in the included studies cont.

|  | **Bahwere *et al.*^S6^** | | | **Sigh *et al.*^S7^** | | **Kohlmann *et al.*^S8^** | | **Hendrixson *et al*.^S9^** | | **Stephenson *et al.*^S10^** | | |
| --- | --- | --- | --- | --- | --- | --- | --- | --- | --- | --- | --- | --- |
|  | **I** | **I** | **C** | **I** | **C** | **I** | **C** | **I** | **C** | **I** | **I** | **C** |
|  | MSMS-RUTF | FSMS-RUTF | PM-RUTF | NumTrey Paste Wafer^g^ | BP 100^h^ | SMS-RUTF | P-RUTF | Oat RUTF | S-RUTF | HO-RUTF | DHA-HO-RUTF | S-RUTF |
| Total energy (kcal/ 100g) | 544 | 532 | 545 | 506 | 529 | 560 | 559 | 537 | 544 |  |  |  |
| Total fat (g/100g) | 36.0 | 34.2 | 33.8 |  |  | 29.2 | 33.0 | 29.8 | 32.6 | 29.5 | 30.2 | 32.7 |
| Fat % of total energy (%) |  |  |  | 49.6 | 51.6 |  |  |  |  |  |  |  |
| SFA (g/100g) | 15.5 | 13.5 | 11.0 |  |  |  |  |  |  |  |  |  |
| MUFA (g/100g) | 12.6 | 11.1 | 18.2 |  |  |  |  |  |  |  |  |  |
| PUFA (g/100g) | 4.09 | 5.6 | 3.2 |  |  |  |  |  |  |  |  |  |
| Total n-6 PUFA (g/100g) |  |  |  |  |  | 6.3 | 5.7 | 5.8 | 8.5 | 2.1 | 2.2 | 5.8 |
| n-6 % of total energy (%) | 3.9 | 5.2 | 5.0 | 15 |  |  |  |  |  |  |  |  |
| Linoleic acid |  |  |  |  |  |  |  |  |  |  |  |  |
| Arachidonic acid |  |  |  |  |  |  |  |  |  |  |  |  |
| Total n-3 PUFA (g/100g) |  |  |  |  |  | 1.9 | 0.03 | 1.4 | 1.6 | 1.4 | 1.5 | 0.5 |
| n-3 % of total energy (%) | 0.2 | 0.4 | 0.5 | 3.6 |  |  |  |  |  |  |  |  |
| Alpha-linolenic acid |  |  |  |  |  |  |  |  |  |  |  |  |
| DHA (mg/100g) |  |  |  |  |  |  |  |  |  | 0 | 72 | 0 |
| EPA (mg/100g) |  |  |  |  |  |  |  |  |  | 0 | 14 | 0 |
| **n-6:n-3 ratio** | 16:1^i^ | 12:1^i^ | 10:1^i^ | 4:1^i^ |  | 3.3:1^i^ | 190:1^i^ | 3.1: 1^i^ | 5.3: 1^i^ | 1.5:1^i^ | 1.5:1^i^ | 11.6:1^i^ |
|  |  |  |  |  |  |  |  |  |  |  |  |  |
| **Sub-group analysis category** | **Higher** n-6: n-3 ratio | | | **RUTF with n-3 LCPUFA** | | **Lower** n-6: n-3 ratio | | **Lower** n-6:n-3 ratio | | **Lower** n-6: n‑3 ratio | **with n-3 LCPUFA**  *Fish oil capsule was added* | |
| **Rational for categorisation** | *Due to addition of soy to the alternative RUTFs* | | | *Due to the n-3 LCPUFA which are naturally found in small indigenous fish* | | *Due to palm and soybean oil replaced with canola oil* | | *Due to high oleic soybeans and palm oil replaced by canola oil* | | *Due to high oleic peanuts and perilla oil* |  |  |

C: comparator; I: intervention; SFA: Saturated fatty acids; MUFA: monounsaturated fatty acids; PUFA: polyunsaturated fatty acids; RUTF: ready to use therapeutic foods; HO-RUTF: high oleic RUTF; S-RUTF: standard RUTF; SMS-RUTF: soya, maize and sorghum RUTF; P-RUTF: peanut-paste RUTF; F-RUTF: flax seed oil-containing RUTF; FFO-RUTF: flax seed oil-containing RUTF with added fish oil capsule; MSMS-RUTF: Milk, soya, maize and sorghum RUTF; FSMS-RUTF: milk-free, soya, maize and sorghum RUTF; n-3: omega 3 PUFA; n-6: omega 6 PUFA; n-3 LCPUFA: omega-3 long-chain polyunsaturated fatty acids; DHA: docosahexaenoic acid.

^a^ Only total fat reported, authors were contacted but no response; ^b^ Reported linoleic acid: alpha-linolenic acid ratio; ^c^ Fatty acid profile for P-RUTF not reported; ^d^ Ingredients of RUTFs used by Jones *et al.*^S4^ and Bahwere *et al*.^S6^ not published and not received from authors upon inquiry; ^e^ Fatty acid profile reported as relative fatty acid content (% of total) reported for linoleic acid, arachidonic acid, alpha-linolenic acid, DHA and EPA; ^f^ Both MSMS-RUTF and FSMS-RUTF were enriched with crystalline amino acids; ^g^Fatty acid profile reported for NumTrey paste; ^h^Fatty acid profile of BP-100 not published; ^i^ n-6: n-3 ratio not reported, but calculated by dividing the reported n-6 by the n-3.

**Table S1 References**

S1. Oakley E, Reinking J, Sandige H, et al. A ready-to-use therapeutic food containing 10% milk is less effective than one with 25% milk in the treatment of severely malnourished children. J Nutr. 2010;140(12):2248-2252.

S2. Hsieh J-C, Liu L, Zeilani M, et al. High oleic ready-to-use therapeutic food maintains docosahexaenoic acid status in severe malnutrition: a randomized, blinded trial. J. Pediatr. Gastroenterol. Nutr. 2015;61(1):138.

S3. Irena AH, Bahwere P, Owino VO, et al. Comparison of the effectiveness of a milk‐free soy‐maize‐sorghum‐based ready‐to‐use therapeutic food to standard ready‐to‐use therapeutic food with 25% milk in nutrition management of severely acutely malnourished Zambian children: an equivalence non‐blinded cluster randomised controlled trial. Matern Child Nutr. 2015;11:105-119.

S4. Jones KD, Ali R, Khasira MA, et al. Ready-to-use therapeutic food with elevated n-3 polyunsaturated fatty acid content, with or without fish oil, to treat severe acute malnutrition: a randomized controlled trial. BMC Med. 2015;13(1):1-14.

S5. Bahwere P, Balaluka B, Wells JC, et al. Cereals and pulse-based ready-to-use therapeutic food as an alternative to the standard milk-and peanut paste–based formulation for treating severe acute malnutrition: a noninferiority, individually randomized controlled efficacy clinical trial. AJCN. 2016;103(4):1145-1161.

S6. Bahwere P, Akomo P, Mwale M, et al. Soya, maize, and sorghum–based ready-to-use therapeutic food with amino acid is as efficacious as the standard milk and peanut paste–based formulation for the treatment of severe acute malnutrition in children: a noninferiority individually randomized controlled efficacy clinical trial in Malawi. AJCN. 2017;106(4):1100-1112.

S7. Sigh S, Roos N, Chamnan C, et al. Effectiveness of a locally produced, fish-based food product on weight gain among Cambodian children in the treatment of acute malnutrition: a randomized controlled trial. Nutrients. 2018;10(7):909.

S8. Kohlmann K, Callaghan-Gillespie M, Gauglitz JM, et al. Alternative ready-to-use therapeutic food yields less recovery than the standard for treating acute malnutrition in children from Ghana. GHSP. 2019;7(2):203-214.

S9. Hendrixson DT, Godbout C, Los A, et al. Treatment of severe acute malnutrition with oat or standard ready-to-use therapeutic food: a triple-blind, randomised controlled clinical trial. Gut. 2020;69(12):2143-2149.

S10. Stephenson K, Callaghan-Gillespie M, Maleta K, et al. Low linoleic acid foods with added DHA given to Malawian children with severe acute malnutrition improves cognition: a randomized, triple blinded, controlled clinical trial. AJCN. 2022;115(5):1322-1333.
