# Supplementary material for: Effect of fatty acid profiles in varying recipes of ready-to-use therapeutic foods on neurodevelopmental and clinical outcomes of children (6–59 months) with severe wasting: a systematic review
Source: Nutr Rev. 2023 Dec 22;82(12):1784–99. doi: 10.1093/nutrit/nuad151 (PMC11551450; doi:10.1093/nutrit/nuad151)
Supplement: nuad151_Supplementary_Data [file nuad151_supplementary_data.zip › nuad151_Supplementary_Data/Table S2 Excluded studies with reasons.docx]

**Table S2 Excluded studies with reasons**

| **Reference** | **Reason for exclusion** |
| --- | --- |
| Bahwere *et al.*  (2014)^S1^ | The study replaced powdered milk in standard RUTF with whey protein concentrate to reduce the cost of the standard RUTF. However, no adjustments to the fatty acid profile were made. |
| Bisimwa *et al.*  (2012)^S2^ | Compared the intervention with complimentary food and not a therapeutic food and the intervention was a corn-soy porridge. |
| Sato *et al.*  (2018)^S3^ | This paper is part of a larger study conducted in Malawi. Although the study is included as well as some of the papers related to the study (Bahwere *et al.,* 2017)^S5^ this paper was excluded as it only provides data that does not fall within the scope of this review. |
| Akomo *et al.*  (2019)^S4^ | This paper is part of a larger study conducted in Malawi. Although the study is included as well as some of the papers related to the study (Bahwere *et al.,* 2017)^S5^ this paper was excluded as it only provides data that does not fall within the scope of this review. |

**Table S2 References**

S1 Bahwere P, Banda T, Sadler K, et al. Effectiveness of milk whey protein-based ready-to-use therapeutic food in treatment of severe acute malnutrition in Malawian under-5 children: a randomised, double-blind, controlled non-inferiority clinical trial. Matern Child Nutr. 2014;10:436-451.

S2 Bisimwa G, Owino VO, Bahwere P. et al. Randomized controlled trial of the effectiveness of a soybean-maize-sorghum-based ready-to-use complementary food paste on infant growth in South Kivu, Democratic Republic of Congo. AJCN. 2012;95(5):1157-1164.

S3 Sato W, Furuta C, Matsunaga K et al. Amino-acid-enriched cereals ready-to-use therapeutic foods (RUTF) are as effective as milk-based RUTF in recovering essential amino acid during the treatment of severe acute malnutrition in children: An individually randomized control trial in Malawi. Plos One, 2018;13(8):e0201686.

S4 Akomo P, Bahwere P, Murakami H et al. Soya, maize and sorghum ready-to-use therapeutic foods are more effective in correcting anaemia and iron deficiency than the standard ready-to-use therapeutic food: randomized controlled trial. BMC Public Health, 2019 19:806.

S5 Bahwere P, Akomo P, Mwale M, et al. Soya, maize, and sorghum–based ready-to-use therapeutic food with amino acid is as efficacious as the standard milk and peanut paste–based formulation for the treatment of severe acute malnutrition in children: a noninferiority individually randomized controlled efficacy clinical trial in Malawi. AJCN. 2017;106(4):1100-1112.
