# Supplementary material for: Effect of fatty acid profiles in varying recipes of ready-to-use therapeutic foods on neurodevelopmental and clinical outcomes of children (6–59 months) with severe wasting: a systematic review
Source: Nutr Rev. 2023 Dec 22;82(12):1784–99. doi: 10.1093/nutrit/nuad151 (PMC11551450; doi:10.1093/nutrit/nuad151)
Supplement: nuad151_Supplementary_Data [file nuad151_supplementary_data.zip › nuad151_Supplementary_Data/Table S3 Summary of findings table and quality.pdf]

Table S3 Summary of findings

Ready-to-use therapeutic foods with different fatty acid profiles in children 6-59 months with severe wasting

Outcomes	Anticipated absolute effects* (95% CI)		Relative effect (95% CI)	№ of participants (studies)	Certainty of the evidence (GRADE)	Comments
	**Risk with standard RUTF**	**Risk with RUTF with different EFA**				
Cognition: Willats problem solving: Problem 1 follow-up: mean 4 weeks			**OR 1.00** (0.73 to 1.35)	871 (2 RCTs)	⨁◯◯◯ Very low[a,b,c]	There was no significant difference in problem-solving skills between the standard RUTF and the alternative RUTFs
Cognition: Willats problem solving: Problem 2 follow-up: mean 4 weeks			**OR 0.94** (0.71 to 1.24)	870 (2 RCTs)	⨁◯◯◯ Very low[a,b,c]	There was no significant difference in problem-solving skills between the standard RUTF and the alternative RUTFs
Cognition: Willats problem solving: Problem 3 follow-up: mean 4 weeks			**OR 1.02** (0.70 to 1.49)	715 (2 RCTs)	⨁◯◯◯ Very low[a,b,c]	There was no significant difference in problem-solving skills between the standard RUTF and the alternative RUTFs
MDAT: Global follow-up: range 5 months to 7 months			-	986 (2 RCTs)	⨁⨁⨁◯ Moderate[a,d,e]	The HO-DHA RUTF group had higher Global MDAT scores compared with the standard RUTF
MDAT: Gross motor follow-up: range 5 months to 7 months			-	985 (2 RCTs)	⨁⨁⨁◯ Moderate[a,d,e]	The HO-DHA RUTF group had higher Gros motor MDAT scores compared with the standard RUTF
MDAT: Fine motor follow-up: range 5 months to 7 months			-	966 (2 RCTs)	⨁⨁⨁◯ Moderate[a,d,e]	There was no difference in fine motor MDAT scores in the alternative RUTFs compared with the standard RUTF
MDAT: Language follow-up: range 5 months to 7 months			-	985 (2 RCTs)	⨁⨁⨁◯ Moderate[a,d,e]	There was no difference in language MDAT scores in the alternative RUTFs compared with the standard RUTF
MDAT: Social follow-up: range 5 months to 7 months			-	985 (2 RCTs)	⨁⨁⨁◯ Moderate[a]	The HO-RUTF and HO-DHA RUTF groups had higher social MDAT scores compared with the standard RUTF
Cognition: Saccade eye-tracking: Infant oriented with attention response time follow-up: range 5 months to 7 months			-	694 (2 RCTs)	⨁⨁◯◯ Low[a,c]	There was no difference in Saccade eye-tracking tests in the alternative RUTFs compared with the standard RUTF

Summary of findings:

Ready-to-use therapeutic foods with different fatty acid profiles in children 6-59 months with severe wasting

Outcomes	**Anticipated absolute effects*** (95% CI)		Relative effect (95% CI)	№ of participants (studies)	Certainty of the evidence (GRADE)	Comments
	**Risk with standard RUTF**	**Risk with RUTF with different EFA**				
Cognition: Saccade eye-tracking: Visual paired comparison novelty preference score follow-up: range 5 months to 7 months			-	767 (2 RCTs)	⊕⊕⊕◯ Moderate[a,e,f]	There was no difference in Saccade eye-tracking tests in the alternative RUTFs compared with the standard RUTF
Cognition: Saccade eye-tracking: Mean fixation duration follow-up: range 5 months to 7 months			-	789 (2 RCTs)	⊕⊕◯◯ Low[a,c]	There was no difference in Saccade eye-tracking tests in the alternative RUTFs compared with the standard RUTF
Plasma fatty acid composition: Linoleic acid follow-up: mean 8 weeks	The mean plasma fatty acid composition: Linoleic acid was **0**	MD **0.83 lower** (1.48 lower to 0.18 lower)	-	646 (3 RCTs)	⊕⊕◯◯ Low[g,h]	There was a significantly higher linoleic acid composition in children receiving standard RUTF compared with children receiving alternative RUTF
Plasma fatty acid composition: Linoleic acid - Lower n-6: n-3 ratio RUTF follow-up: mean 8 weeks	The mean plasma fatty acid composition: Linoleic acid - Lower n-6: n-3 ratio RUTF was **0**	MD **0.73 lower** (1.61 lower to 0.14 higher)	-	384 (2 RCTs)	⊕◯◯◯ Very low[b,g,h]	There was no significant difference in linoleic acid composition in children receiving standard RUTF compared with children receiving alternative RUTF
Plasma fatty acid composition: Linoleic acid - RUTF with n-3 LCPUFA follow-up: mean 8 weeks	The mean plasma fatty acid composition: Linoleic acid - RUTF with n-3 LCPUFA was **0**	MD **0.95 lower** (1.91 lower to 0.01 higher)	-	262 (1 RCT)	⊕◯◯◯ Very low[b,g,h]	There was a borderline significantly higher linoleic acid composition in children receiving standard RUTF compared with children receiving alternative RUTF with n-3 LCPUFA
Plasma fatty acid composition: Alpha-Linolenic acid follow-up: mean 8 weeks	The mean plasma fatty acid composition: Alpha-Linolenic acid was **0**	MD **0.23 higher** (0.18 higher to 0.28 higher)	-	646 (3 RCTs)	⊕⊕◯◯ Low[g,h]	Children receiving alternative formulations of RUTF had a higher plasma phospholipid ALA composition compared with those receiving the standard RUTF
Plasma fatty acid composition: Alpha-Linolenic acid - Lower n-6: n-3 ratio RUTF follow-up: mean 8 weeks	The mean plasma fatty acid composition: Alpha-Linolenic acid - Lower n-6: n-3 ratio RUTF was **0**	MD **0.21 higher** (0.14 higher to 0.29 higher)	-	384 (2 RCTs)	⊕◯◯◯ Very low[b,g,h]	Children receiving alternative RUTF formulations with a lower n-6: n-3 PUFA ratio had a higher plasma phospholipid ALA composition compared with those receiving the standard RUTF

Summary of findings:

Ready-to-use therapeutic foods with different fatty acid profiles in children 6-59 months with severe wasting

Outcomes	Anticipated absolute effects* (95% CI)		Relative effect (95% CI)	№ of participants (studies)	Certainty of the evidence (GRADE)	Comments
	**Risk with standard RUTF**	**Risk with RUTF with different EFA**				
Plasma fatty acid composition: Alpha-Linolenic acid - RUTF with n-3 LCPUFA follow-up: mean 8 weeks	The mean plasma fatty acid composition: Alpha-Linolenic acid - RUTF with n-3 LCPUFA was **0**	MD **0.25 higher** (0.17 higher to 0.33 higher)	-	262 (1 RCT)	⊕◯◯◯ Very low[b,g,h]	Children receiving alternative RUTF formulations with n-3 LCPUFA had a higher plasma phospholipid ALA composition compared with those receiving the standard RUTF
Plasma fatty acid composition: Arachidonic acid follow-up: mean 8 weeks	The mean plasma fatty acid composition: Arachidonic acid was **0**	MD **0.73 lower** (1.22 lower to 0.24 lower)	-	646 (3 RCTs)	⊕⊕◯◯ Low[g,h,i]	Children receiving alternative RUTF had lower plasma phospholipid arachidonic acid compositions compared with children receiving standard RUTF
Plasma fatty acid composition: Arachidonic acid - Lower n-6: n-3 ratio RUTF follow-up: mean 8 weeks	The mean plasma fatty acid composition: Arachidonic acid - Lower n-6: n-3 ratio RUTF was **0**	MD **1.26 lower** (1.9 lower to 0.61 lower)	-	384 (2 RCTs)	⊕◯◯◯ Very low[b,g,h,i]	Children receiving alternative RUTF had lower plasma phospholipid arachidonic acid compositions compared with children receiving standard RUTF
Plasma fatty acid composition: Arachidonic acid - RUTF with n-3 LCPUFA follow-up: mean 8 weeks	The mean plasma fatty acid composition: Arachidonic acid - RUTF with n-3 LCPUFA was **0**	MD **0** (0.75 lower to 0.75 higher)	-	262 (1 RCT)	⊕◯◯◯ Very low[b,g,h]	There was no difference in plasma phospholipid arachidonic acid compositions between children receiving alternative RUTFs with n-3 LCPUFA and those receiving the standard RUTF
Plasma fatty acid composition: Eicosapentaenoic acid follow-up: mean 8 weeks	The mean plasma fatty acid composition: Eicosapentaenoic acid was **0**	MD **0.2 higher** (0.15 higher to 0.25 higher)	-	646 (3 RCTs)	⊕⊕◯◯ Low[g,h,i]	Children receiving alternative formulations of RUTF had higher plasma phospholipid EPA compositions compared to those receiving standard RUTF
Plasma fatty acid composition: Eicosapentaenoic acid - Lower n-6: n-3 ratio RUTF follow-up: mean 8 weeks	The mean plasma fatty acid composition: Eicosapentaenoic acid - Lower n-6: n-3 ratio RUTF was **0**	MD **0.18 higher** (0.11 higher to 0.24 higher)	-	384 (2 RCTs)	⊕◯◯◯ Very low[b,g,h,i]	Children receiving alternative formulations of RUTF with a lower n-6: n-3 PUFA ratio had higher plasma phospholipid EPA compositions compared to those receiving standard RUTF
Plasma fatty acid composition: Eicosapentaenoic acid - RUTF with n-3 LCPUFA follow-up: mean 8 weeks	The mean plasma fatty acid composition: Eicosapentaenoic acid - RUTF with n-3 LCPUFA was **0**	MD **0.24 higher** (0.16 higher to 0.32 higher)	-	262 (1 RCT)	⊕◯◯◯ Very low[b,g,h]	Children receiving RUTF with n-3 LCPUFA had higher plasma phospholipid EPA compositions compared to those receiving standard RUTF

Summary of findings:

Ready-to-use therapeutic foods with different fatty acid profiles in children 6-59 months with severe wasting

Outcomes	Anticipated absolute effects* (95% CI)		Relative effect (95% CI)	№ of participants (studies)	Certainty of the evidence (GRADE)	Comments
	**Risk with standard RUTF**	**Risk with RUTF with different EFA**				
Plasma fatty acid composition: Docosapentaenoic acid omega-6 follow-up: mean 8 weeks	The mean plasma fatty acid composition: Docosapentaenoic acid omega-6 was **0**	MD **0.07 lower** (0.11 lower to 0.04 lower)	-	505 (2 RCTs)	⨁⨁⨁⨁ High	The plasma phospholipid n-6 docosapentaenoic acid composition (assessed in 1 study) was higher in children receiving the standard RUTF compared with the two arms of lower n-6: n-3 PUFA ratio RUTF and RUTF with n-3 LCPUFA
Plasma fatty acid composition: Docosapentaenoic acid omega-6 - Lower n-6: n-3 ratio RUTF follow-up: mean 8 weeks	The mean plasma fatty acid composition: Docosapentaenoic acid omega-6 - Lower n-6: n-3 ratio RUTF was **0**	MD **0.06 lower** (0.11 lower to 0.01 lower)	-	243 (1 RCT)	⨁⨁⨁◯ Moderate[b]	The plasma phospholipid n-6 docosapentaenoic acid composition (assessed in 1 study) was higher in children receiving the standard RUTF compared
Plasma fatty acid composition: Docosapentaenoic acid omega-6 - RUTF with n-3 LCPUFA follow-up: mean 8 weeks	The mean plasma fatty acid composition: Docosapentaenoic acid omega-6 - RUTF with n-3 LCPUFA was **0**	MD **0.08 lower** (0.13 lower to 0.03 lower)	-	262 (1 RCT)	⨁⨁⨁◯ Moderate[b]	The plasma phospholipid n-6 docosapentaenoic acid composition (assessed in 1 study) was higher in children receiving the standard RUTF compared with the two arms of lower n-6: n-3 PUFA ratio RUTF and RUTF with n-3 LCPUFA
Plasma fatty acid composition: Docosahexaenoic acid follow-up: mean 8 weeks	The mean plasma fatty acid composition: Docosahexaenoic acid was **0**	MD **0.33 higher** (0.15 higher to 0.5 higher)	-	862 (3 RCTs)	⨁⨁◯◯ Low[g,h,i]	Children receiving alternative RUTFs with an altered fatty acid profile had a higher plasma phospholipid DHA composition compared with those receiving the standard RUTF
Plasma fatty acid composition: Docosahexaenoic acid - Lower n-6: n-3 RUTF follow-up: mean 8 weeks	The mean plasma fatty acid composition: Docosahexaenoic acid - Lower n-6: n-3 RUTF was **0**	MD **0.07 higher** (0.17 lower to 0.3 higher)	-	492 (2 RCTs)	⨁◯◯◯ Very low[b,g,h,i]	Children receiving RUTFs with a lower n-6: n-3 PUFA ratio did not have significantly different plasma phospholipid DHA compositions compared with those receiving the standard RUTF
Plasma fatty acid composition: Docosahexaenoic acid - RUTF with n-3 LCPUFA follow-up: mean 8 weeks	The mean plasma fatty acid composition: Docosahexaenoic acid - RUTF with n-3 LCPUFA was **0**	MD **0.66 higher** (0.39 higher to 0.93 higher)	-	370 (1 RCT)	⨁◯◯◯ Very low[b,g,h]	Children receiving RUTF with n-3 LCPUFA had a higher plasma phospholipid DHA composition compared with those receiving the standard RUTF
Nutrition recovery and growth: Rate of weight gain follow-up: range 4 weeks to 12 weeks	The mean nutrition recovery and growth: The rate of weight gain was **0**	MD **0.15 lower** (0.67 lower to 0.37 higher)	-	7468 (8 RCTs)	⨁⨁◯◯ Low[i,j,k,l,m]	The overall rate of weight gain was not significantly different between the children receiving alternative RUTFs and those receiving standard RUTFs

Summary of findings:

Ready-to-use therapeutic foods with different fatty acid profiles in children 6-59 months with severe wasting

Outcomes	**Anticipated absolute effects*** (95% CI)		Relative effect (95% CI)	№ of participants (studies)	Certainty of the evidence (GRADE)	Comments
	**Risk with standard RUTF**	**Risk with RUTF with different EFA**				
Nutrition recovery and growth: Rate of weight gain - Lower n-6: n-3 ratio RUTF follow-up: range 4 weeks to 12 weeks	The mean nutrition recovery and growth: Rate of weight gain - Lower n-6: n-3 ratio RUTF was **0**	MD **0.04 lower** (0.87 lower to 0.79 higher)	-	4296 (5 RCTs)	⨁⨁◯◯ Low[i,j,l]	The mean rate of weight gain was not significantly different between the children receiving the RUTFs with a lower n-6: n-3 profile compared with those receiving a standard RUTF
Nutrition recovery and growth: Rate of weight gain - RUTF with n-3 LCPUFA follow-up: range 4 weeks to 12 weeks	The mean nutrition recovery and growth: Rate of weight gain - RUTF with n-3 LCPUFA was **0**	MD **0.25 lower** (0.64 lower to 0.15 higher)	-	1298 (2 RCTs)	⨁⨁⨁⨁ High[k]	The mean rate of weight gain was not significantly different between the children receiving the RUTFs with n-3 LCPUFA compared with those receiving a standard RUTF
Nutrition recovery and growth: Rate of weight gain - Higher n-6: n-3 ratio RUTF follow-up: range 4 weeks to 12 weeks	The mean nutrition recovery and growth: Rate of weight gain - Higher n-6: n-3 ratio RUTF was **0**	MD **0.5 lower** (0.75 lower to 0.25 lower)	-	1874 (1 RCT)	⨁⨁◯◯ Low[m,n]	The mean rate of weight gain was lower in children (from 1 study) receiving RUTF with a higher n-6: n-3 PUFA ratio compared with those receiving the standard RUTF
Nutrition recovery and growth: Rate of MUAC gain follow-up: range 4 weeks to 12 weeks	The mean nutrition recovery and growth: Rate of MUAC gain was **0**	MD **0.01 lower** (0.05 lower to 0.02 higher)	-	6393 (7 RCTs)	⨁⨁◯◯ Low[i,k,l]	The overall rate of MUAC gain was not significantly different between the children receiving alternative RUTFs and those receiving standard RUTFs
Nutrition recovery and growth: Rate of MUAC gain - Lower n-6: n-3 ratio RUTF follow-up: range 4 weeks to 12 weeks	The mean nutrition recovery and growth: Rate of MUAC gain - Lower n-6: n-3 ratio RUTF was **0**	MD **0.01 higher** (0.06 lower to 0.07 higher)	-	3222 (4 RCTs)	⨁⨁◯◯ Low[i,l]	The mean rate of MUAC gain was not significantly different between the children receiving the RUTFs with a lower n-6: n-3 profile compared with those receiving a standard RUTF
Nutrition recovery and growth: Rate of MUAC gain - Higher n-6: n-3 ratio RUTF follow-up: range 4 weeks to 12 weeks	The mean nutrition recovery and growth: Rate of MUAC gain - Higher n-6: n-3 ratio RUTF was **0**	MD **0.04 lower** (0.06 lower to 0.02 lower)	-	1874 (1 RCT)	⨁⨁⨁◯ Moderate[n]	The mean rate of MUAC gain was lower in children (from 1 study) receiving RUTF with a higher n-6: n-3 PUFA ratio compared with those receiving the standard RUTF
Nutrition recovery and growth: Rate of MUAC gain - RUTF with n-3 LCPUFA follow-up: range 4 weeks to 12 weeks	The mean nutrition recovery and growth: Rate of MUAC gain - RUTF with n-3 LCPUFA was **0**	MD **0.03 lower** (0.05 lower to 0)	-	1297 (2 RCTs)	⨁⨁⨁⨁ High[k]	The mean rate of MUAC gain was lower in children receiving the RUTFs with n-3 LCPUFA compared with those receiving a standard RUTF

Summary of findings:

Ready-to-use therapeutic foods with different fatty acid profiles in children 6-59 months with severe wasting

Outcomes	Anticipated absolute effects* (95% CI)		Relative effect (95% CI)	№ of participants (studies)	Certainty of the evidence (GRADE)	Comments
	**Risk with standard RUTF**	**Risk with RUTF with different EFA**				
Nutrition recovery and growth: Rate of length gain follow-up: range 4 weeks to 12 weeks	The mean nutrition recovery and growth: Rate of length gain was **0**	MD **0** (0.04 lower to 0.05 higher)	-	4509 (4 RCTs)	⊕⊕◯◯ Low[i,l]	The overall rate of height/ length gain was not significantly different between the children receiving alternative RUTFs and those receiving standard RUTFs
Nutrition recovery and growth: Rate of length gain - Lower n-6: n-3 ratio RUTF follow-up: range 4 weeks to 12 weeks	The mean nutrition recovery and growth: Rate of length gain - Lower n-6: n-3 ratio RUTF was **0**	MD **0.03 higher** (0.04 lower to 0.1 higher)	-	1413 (2 RCTs)	⊕⊕⊕◯ Moderate[l]	The mean rate of height/ length gain was not significantly different between the children receiving the RUTFs with a lower n-6: n-3 profile compared with those receiving a standard RUTF
Nutrition recovery and growth: Rate of length gain - Higher n-6: n-3 ratio RUTF follow-up: range 4 weeks to 12 weeks	The mean nutrition recovery and growth: Rate of length gain - Higher n-6: n-3 ratio RUTF was **0**	MD **0.04 lower** (0.06 lower to 0.02 lower)	-	1874 (1 RCT)	⊕⊕⊕◯ Moderate[n]	The mean rate of height/ length gain was lower in children (from 1 study) receiving RUTF with a higher n-6: n-3 PUFA ratio compared with those receiving the standard RUTF
Nutrition recovery and growth: Rate of length gain - RUTF with n-3 LCPUFA follow-up: range 4 weeks to 12 weeks	The mean nutrition recovery and growth: Rate of length gain - RUTF with n-3 LCPUFA was **0**	MD **0.02 higher** (0.02 lower to 0.06 higher)	-	1222 (1 RCT)	⊕⊕⊕⊕ High	The mean rate of length/ height gain was not significantly different in children receiving the RUTFs with n-3 LCPUFA compared with those receiving a standard RUTF
Nutrition recovery and growth: Recovered follow-up: range 4 weeks to 12 weeks	714 per 1,000	**685 per 1,000** (643 to 726)	**OR 0.87** (0.72 to 1.06)	10934 (10 RCTs)	⊕⊕⊕◯ Moderate[i]	There was no significant difference in the odds of recovery between the children receiving the standard RUTFs compared with those receiving alternative RUTFs
Nutrition recovery and growth: Recovered - Lower n-6: n-3 ratio RUTF follow-up: range 4 weeks to 12 weeks	646 per 1,000	**605 per 1,000** (523 to 683)	**OR 0.84** (0.60 to 1.18)	6058 (6 RCTs)	⊕⊕⊕◯ Moderate[i]	There was no significant difference in the odds of recovery between the children receiving the standard RUTFs compared with those receiving alternative RUTFs with a lower n-6: n-3 PUFA ratio
Nutrition recovery and growth: Recovered - Higher n-6: n-3 ratio RUTF follow-up: range 4 weeks to 12 weeks	814 per 1,000	**800 per 1,000** (772 to 824)	**OR 0.91** (0.77 to 1.07)	3619 (3 RCTs)	⊕⊕⊕◯ Moderate[o]	There was no significant difference in the odds of recovery between the children receiving the standard RUTFs compared with those receiving alternative RUTFs with a higher n-6: n-3 PUFA ratio

Summary of findings:

Ready-to-use therapeutic foods with different fatty acid profiles in children 6-59 months with severe wasting

Outcomes	Anticipated absolute effects* (95% CI): Risk with standard RUTF	Anticipated absolute effects* (95% CI): Risk with RUTF with different EFA	Relative effect (95% CI)	№ of participants (studies)	Certainty of the evidence (GRADE)	Comments
Nutrition recovery and growth: Recovered - RUTF with n-3 LCPUFA follow-up: range 4 weeks to 12 weeks	750 per 1,000	**721 per 1,000** (664 to 771)	**OR 0.86** (0.66 to 1.12)	1257 (1 RCT)	⨁⨁⨁⨁ High	There was no significant difference in the odds of recovery between the children receiving the standard RUTFs compared with those receiving alternative RUTFs with n-3 LCPUFA
Nutrition recovery and growth: WHZ- follow-up: range 4 weeks to 12 weeks	The mean nutrition recovery and growth: WHZ was **0**	MD **0.12 higher** (0 to 0.25 higher)	-	2145 (4 RCTs)	⨁⨁⨁◯ Moderate[i]	The random-effects pooled estimate showed a borderline significant overall mean difference in favour of the alternative RUTF concerning WHZ
Nutrition recovery and growth: WHZ - Lower n-6: n-3 ratio RUTF follow-up: range 4 weeks to 12 weeks	The mean nutrition recovery and growth: WHZ - Lower n-6: n-3 ratio RUTF was **0**	MD **0.4 higher** (0.11 higher to 0.69 higher)	-	196 (2 RCTs)	⨁⨁⨁◯ Moderate[b]	The children receiving alternative RUTFs with a lower n-6: n-3 PUFA ratio had a higher WHZ compared with those receiving standard RUTFs
Nutrition recovery and growth: WHZ - RUTF with n-3 LCPUFA follow-up: range 4 weeks to 12 weeks	The mean nutrition recovery and growth: WHZ - RUTF with n-3 LCPUFA was **0**	MD **0.13 higher** (0.09 higher to 0.17 higher)	-	75 (1 RCT)	⨁⨁⨁◯ Moderate[b]	The children receiving alternative RUTFs with n-3 LCPUFA had a higher WHZ compared with those receiving standard RUTFs (1 study)
Nutrition recovery and growth: WHZ - Higher n-6: n-3 ratio RUTF follow-up: range 4 weeks to 12 weeks	The mean nutrition recovery and growth: WHZ - Higher n-6: n-3 ratio RUTF was **0**	MD **0** (0.1 lower to 0.1 higher)	-	1874 (1 RCT)	⨁⨁⨁⨁ High	There was no significant difference in the WHZ-score of children receiving alternative RUTFs with a higher n-6: n-3 PUFA ratio compared with those receiving standard RUTFs
Nutrition recovery and growth: HAZ follow-up: range 4 weeks to 12 weeks	The mean nutrition recovery and growth: HAZ was **0**	MD **0.11 lower** (0.25 lower to 0.02 higher)	-	1929 (2 RCTs)	⨁⨁⨁⨁ High	There was no significant difference in the HAZ-score between children receiving alternative RUTFs and those receiving standard RUTFs
Nutrition recovery and growth: HAZ - Lower n-6: n-3 ratio RUTF follow-up: range 4 weeks to 12 weeks	The mean nutrition recovery and growth: HAZ - Lower n-6: n-3 ratio RUTF was **0**	MD **0.44 lower** (1.08 lower to 0.2 higher)	-	55 (1 RCT)	⨁⨁⨁◯ Moderate[b]	There was no significant difference in the HAZ between children receiving alternative RUTFs with a lower n-6: n-3 PUFA and those receiving standard RUTFs
Nutrition recovery and growth: HAZ - Higher n-6: n-3 ratio RUTF follow-up: range 4 weeks to 12 weeks	The mean nutrition recovery and growth: HAZ - Higher n-6: n-3 ratio RUTF was **0**	MD **0.1 lower** (0.24 lower to 0.04 higher)	-	1874 (1 RCT)	⨁⨁⨁⨁ High	There was no significant difference in the HAZ between children receiving alternative RUTFs with a higher n-6: n-3 PUFA and those receiving standard RUTFs
Nutrition recovery and growth: WAZ-score follow-up: range 4 weeks to 12 weeks	The mean nutrition recovery and growth: WAZ-score was **0**	MD **0.1 lower** (0.2 lower to 0.01 higher)	-	1929 (2 RCTs)	⨁⨁⨁⨁ High	There was no significant difference in the WAZ-score between children receiving alternative RUTFs and those receiving standard RUTFs

Summary of findings:

Ready-to-use therapeutic foods with different fatty acid profiles in children 6-59 months with severe wasting

Outcomes	Anticipated absolute effects[*] (95% CI)		Relative effect (95% CI)	№ of participants (studies)	Certainty of the evidence (GRADE)	Comments
	**Risk with standard RUTF**	**Risk with RUTF with different EFA**				
Nutrition recovery and growth: WAZ - Lower n-6: n-3 ratio RUTF follow-up: range 4 weeks to 12 weeks	The mean nutrition recovery and growth: WAZ - Lower n-6: n-3 ratio RUTF was **0**	MD **0.04 lower** (0.5 lower to 0.42 higher)	-	55 (1 RCT)	⊕⊕⊕◯ Moderate[b]	There was no significant difference in the WAZ between children receiving alternative RUTFs with a lower n-6: n-3 PUFA and those receiving standard RUTFs
Nutrition recovery and growth: WAZ - Higher n-6: n-3 ratio RUTF follow-up: range 4 weeks to 12 weeks	The mean nutrition recovery and growth: WAZ - Higher n-6: n-3 ratio RUTF was **0**	MD **0.1 lower** (0.21 lower to 0.01 higher)	-	1874 (1 RCT)	⊕⊕⊕⊕ High	There was no significant difference in the WAZ between children receiving alternative RUTFs with a higher n-6: n-3 PUFA and those receiving standard RUTFs
Mortality rate follow-up: range 4 weeks to 12 weeks	56 per 1,000	**44 per 1,000** (37 to 52)	**RR 0.79** (0.67 to 0.94)	10116 (12 RCTs)	⊕⊕⊕⊕ High	There was a higher risk of mortality in children on standard RUTFs compared with those receiving the alternative RUTFs
Mortality rate - Lower n-6: n-3 ratio RUTF follow-up: range 4 weeks to 12 weeks	78 per 1,000	**56 per 1,000** (46 to 68)	**RR 0.72** (0.59 to 0.87)	5656 (7 RCTs)	⊕⊕⊕⊕ High	There was a higher risk of mortality in children on standard RUTFs compared with those receiving the alternative RUTFs with a lower n-6: n-3 PUFA ratio
Mortality rate - Higher n-6: n-3 ratio RUTF follow-up: range 4 weeks to 12 weeks	29 per 1,000	**29 per 1,000** (19 to 45)	**RR 1.02** (0.67 to 1.57)	3173 (3 RCTs)	⊕⊕⊕◯ Moderate[c]	There was no significant difference in the risk of mortality in children on standard RUTFs compared with those receiving the alternative RUTFs with a higher n-6: n-3 PUFA ratio
Mortality rate - RUTF with n-3 LCPUFA follow-up: range 4 weeks to 12 weeks	17 per 1,000	**25 per 1,000** (11 to 56)	**RR 1.44** (0.64 to 3.21)	1287 (2 RCTs)	⊕⊕⊕◯ Moderate[c]	There was no significant difference in the risk of mortality in children on standard RUTFs compared with those receiving the alternative RUTFs with n-3 LCPUFA
Comorbidities: Fever follow-up: range 4 weeks to 12 weeks	455 per 1,000	**428 per 1,000** (400 to 459)	**RR 0.94** (0.88 to 1.01)	3811 (3 RCTs)	⊕⊕⊕◯ Moderate[i]	There was no significant difference in reported fever between children receiving alternative RUTFs and those receiving standard RUTFs
Comorbidities: Fever - Lower n-6: n-3 ratio RUTF follow-up: range 4 weeks to 12 weeks	515 per 1,000	**494 per 1,000** (458 to 535)	**RR 0.96** (0.89 to 1.04)	2589 (2 RCTs)	⊕⊕⊕◯ Moderate[i]	There was no significant difference in reported fever between children receiving alternative RUTFs with a lower n-6: n-3 PUFA ratio and those receiving standard RUTFs
Comorbidities: Fever - RUTF with n-3 LCPUFA follow-up: range 4 weeks to 12 weeks	307 per 1,000	**267 per 1,000** (221 to 319)	**RR 0.87** (0.72 to 1.04)	1222 (1 RCT)	⊕⊕⊕◯ Moderate[b]	There was no significant difference in reported fever between children receiving alternative RUTFs with n-3 LCPUFA and those receiving standard RUTFs

Summary of findings:

Ready-to-use therapeutic foods with different fatty acid profiles in children 6-59 months with severe wasting

Outcomes	Anticipated absolute effects[*] (95% CI)		Relative effect (95% CI)	№ of participants (studies)	Certainty of the evidence (GRADE)	Comments
	**Risk with standard RUTF**	**Risk with RUTF with different EFA**				
Comorbidities: Diarrhoea follow-up: range 4 weeks to 12 weeks	274 per 1,000	**257 per 1,000** (233 to 287)	**RR 0.94** (0.85 to 1.05)	3867 (5 RCTs)	⊕⊕⊕⊕ High	There was no significant difference in diarrhoea reported between children receiving alternative RUTFs and those receiving standard RUTFs
Comorbidities: Diarrhoea - Lower n-6: n-3 ratio RUTF follow-up: range 4 weeks to 12 weeks	248 per 1,000	**238 per 1,000** (209 to 273)	**RR 0.96** (0.84 to 1.10)	2616 (3 RCTs)	⊕⊕⊕⊕ High	There was no significant difference in diarrhoea reported between children receiving alternative RUTFs with a lower n-6: n-3 PUFA ratio and those receiving standard RUTFs
Comorbidities: Diarrhoea - RUTF with n-3 LCPUFA follow-up: range 4 weeks to 12 weeks	336 per 1,000	**305 per 1,000** (258 to 362)	**RR 0.91** (0.77 to 1.08)	1251 (2 RCTs)	⊕⊕⊕◯ Moderate[b]	There was no significant difference in diarrhoea reported between children receiving alternative RUTFs with n-3 LCPUFA and those receiving standard RUTFs
Comorbidities: LRTI follow-up: range 4 weeks to 12 weeks	150 per 1,000	**150 per 1,000** (42 to 545)	**RR 1.00** (0.28 to 3.63)	60 (2 RCTs)	⊕⊕◯◯ Low[b,c]	There was no significant difference in LRTIs reported between children receiving alternative RUTFs and those receiving standard RUTFs
Comorbidities: LRTI - Lower n-6: n-3 ratio RUTF follow-up: range 4 weeks to 12 weeks	200 per 1,000	**100 per 1,000** (16 to 610)	**RR 0.50** (0.08 to 3.05)	30 (1 RCT)	⊕⊕◯◯ Low[b,c]	There was no significant difference in LRTIs reported between children receiving alternative RUTFs with a lower n-6: n-3 PUFA ratio and those receiving standard RUTFs
Comorbidities: LRTI - RUTF with n-3 LCPUFA follow-up: range 4 weeks to 12 weeks	100 per 1,000	**200 per 1,000** (26 to 1,000)	**RR 2.00** (0.26 to 15.62)	30 (1 RCT)	⊕⊕◯◯ Low[b,c]	There was no significant difference in LRTIs reported between children receiving alternative RUTFs with n-3 LCPUFA and those receiving standard RUTFs
Comorbidities: URTI follow-up: range 4 weeks to 12 weeks	350 per 1,000	**875 per 1,000** (476 to 1,000)	**RR 2.50** (1.36 to 4.58)	60 (2 RCTs)	⊕⊕◯◯ Low[b,c]	A statistically significant difference in URTIs with a higher risk of URTIs in children receiving standard RUTFs compared with those receiving alternative RUTFs (only 1 study)
Comorbidities: URTI - Lower n-6: n-3 ratio RUTF follow-up: range 4 weeks to 12 weeks	400 per 1,000	**952 per 1,000** (440 to 1,000)	**RR 2.38** (1.10 to 5.11)	30 (1 RCT)	⊕⊕◯◯ Low[b,c]	A statistically significant difference in URTIs with a higher risk of URTIs in children on standard RUTFs compared with those receiving alternative RUTFs with a lower n-6: n-3 PUFA ratio (only 1 study)
Comorbidities: URTI - RUTF with n-3 LCPUFA follow-up: range 4 weeks to 12 weeks	300 per 1,000	**801 per 1,000** (303 to 1,000)	**RR 2.67** (1.01 to 7.05)	30 (1 RCT)	⊕⊕◯◯ Low[b,c]	There was a trend towards a statistically significant difference (P = 0.05) in URTIs with a higher risk of URTIs in children receiving standard RUTF compared with those receiving alternative RUTFs with n-3 LCPUFA (only 1 study)

Summary of findings:

Ready-to-use therapeutic foods with different fatty acid profiles in children 6-59 months with severe wasting

Outcomes	Anticipated absolute effects* (95% CI): Risk with standard RUTF	Anticipated absolute effects* (95% CI): Risk with RUTF with different EFA	Relative effect (95% CI)	№ of participants (studies)	Certainty of the evidence (GRADE)	Comments
Comorbidities: Rash follow-up: range 4 weeks to 12 weeks	500 per 1,000	**451 per 1,000** (219 to 706)	**OR 0.82** (0.28 to 2.40)	60 (3 RCTs)	⨁◯◯◯ Very low[b,c,i]	There was no significant difference in rash reported between children receiving alternative RUTFs and those receiving standard RUTFs
Comorbidities: Rash - Lower n-6: n-3 ratio RUTF follow-up: range 4 weeks to 12 weeks	500 per 1,000	**248 per 1,000** (65 to 623)	**OR 0.33** (0.07 to 1.65)	30 (2 RCTs)	⨁⨁◯◯ Low[b,c]	There was no significant difference in rash reported between children receiving alternative RUTFs with a lower n-6: n-3 PUFA ratio and those receiving standard RUTFs
Comorbidities: Rash - RUTF with n-3 LCPUFA follow-up: range 4 weeks to 12 weeks	500 per 1,000	**650 per 1,000** (286 to 897)	**OR 1.86** (0.40 to 8.69)	30 (1 RCT)	⨁⨁◯◯ Low[b,c]	There was no significant difference in rash reported between children receiving alternative RUTFs with n-3 LCPUFA and those receiving standard RUTFs
Comorbidities: Cough follow-up: range 4 weeks to 12 weeks	537 per 1,000	**565 per 1,000** (511 to 619)	**OR 1.12** (0.90 to 1.40)	1315 (1 RCT)	⨁⨁⨁⨁ High	There was no significant difference in cough reported between children receiving alternative RUTFs and those receiving standard RUTFs (1 study)
Adverse events: Vomiting follow-up: range 4 weeks to 12 weeks	114 per 1,000	**134 per 1,000** (101 to 177)	**OR 1.20** (0.87 to 1.66)	1375 (3 RCTs)	⨁⨁⨁⨁ High	There was no significant difference in vomiting reported between children receiving alternative RUTFs and those receiving standard RUTFs
Adverse events: Vomiting - Lower n-6: n-3 ratio RUTF follow-up: range 4 weeks to 12 weeks	115 per 1,000	**131 per 1,000** (97 to 172)	**OR 1.16** (0.83 to 1.60)	1345 (2 RCTs)	⨁⨁⨁⨁ High	There was no significant difference in rash reported between children receiving alternative RUTFs with a lower n-6: n-3 PUFA ratio and those receiving standard RUTFs
Adverse events: Vomiting - RUTF with n-3 LCPUFA follow-up: range 4 weeks to 12 weeks	100 per 1,000	**350 per 1,000** (54 to 838)	**OR 4.85** (0.51 to 46.49)	30 (1 RCT)	⨁⨁◯◯ Low[b,c]	There was no significant difference in rash reported between children receiving alternative RUTFs with n-3 LCPUFA and those receiving standard RUTFs

***The risk in the intervention group** (and its 95% confidence interval) is based on the assumed risk in the comparison group and the **relative effect** of the intervention (and its 95% CI).

CI: confidence interval; **MD:** mean difference; **OR:** odds ratio; **RR:** risk ratio

GRADE Working Group grades of evidence
High certainty: we are very confident that the true effect lies close to that of the estimate of the effect.
Moderate certainty: we are moderately confident in the effect estimate: the true effect is likely to be close to the estimate of the effect, but there is a possibility that it is substantially different.
Low certainty: our confidence in the effect estimate is limited: the true effect may be substantially different from the estimate of the effect.
Very low certainty: we have very little confidence in the effect estimate: the true effect is likely to be substantially different from the estimate of effect.

Explanations

a. The outcome results were based on only one study (Stephenson[S1]) with two study arms. Although the study design fit the PICO for the review, results should be interpreted with caution since it was only one study conducted in one country (Malawi) and thus results might not be generalisable to other countries and populations.

b. Sample size for the outcome was small which reduced the power of analysis.

c. The CI was wide and included the null effect. Thus no conclusion can be made regarding benefit.

d. The sample size was large enough to allow for sufficient power of analyses

e. CI was narrow and thus considered acceptable.

f. Heterogeneity between the two arms was 45%, however, it was decided that the cut-off value for acceptable heterogeneity was 50%, thus this was considered not serious.

g. One study (Jones[S2]) could not be pooled with the rest of the data as they measured erythrocyte fatty acid composition instead of plasma composition. Results were reported as median and IQR.

h. Duration of the intervention varied.

i. Heterogeneity was more than 50% and thus high.

j. Irena[S3] was judged with some concern for bias due to the high risk of bias relating to randomization and deviation of intended deviation.

k. Sigh[S4] was judged with some concern for bias due to some concerns arising from deviation of intended interventions and high risk of bias in the measurement of outcomes.

l. Bahwere[S5] could not be pooled as data were reported per age group.

m. Bahwere[S6] could not be pooled as the data were reported per age group.

n. Only one study, namely Oakley[S7]. This study also did not quite meet the review PICO statement as it did not report the fatty acid profile of the alternative RUTF.

o. One of the included studies, Oakley[S7], did not quite meet the review PICO statement as it did not report the fatty acid profile of the alternative RUTF.

Table S2 References

S1 Stephenson K, Callaghan-Gillespie M, Maleta K, et al. Low linoleic acid foods with added DHA given to Malawian children with severe acute malnutrition improves cognition: a randomized, triple blinded, controlled clinical trial. AJCN. 2022;115(5):1322-1333.

S2 Jones KD, Ali R, Khasira MA, et al. Ready-to-use therapeutic food with elevated n-3 polyunsaturated fatty acid content, with or without fish oil, to treat severe acute malnutrition: a randomized controlled trial. BMC Med. 2015;13(1):1-14.

S3 Irena AH, Bahwere P, Owino VO, et al. Comparison of the effectiveness of a milk‐free soy‐maize‐sorghum‐based ready‐to‐use therapeutic food to standard ready‐to‐use therapeutic food with 25% milk in nutrition management of severely acutely malnourished Zambian children: an equivalence non‐blinded cluster randomised controlled trial. Matern Child Nutr. 2015;11:105-119.

S4 Sigh S, Roos N, Chamnan C, et al. Effectiveness of a locally produced, fish-based food product on weight gain among Cambodian children in the treatment of acute malnutrition: a randomized controlled trial. Nutrients. 2018;10(7):909.

S5 Bahwere P, Balaluka B, Wells JC, et al. Cereals and pulse-based ready-to-use therapeutic food as an alternative to the standard milk-and peanut paste–based formulation for treating severe acute malnutrition: a noninferiority, individually randomized controlled efficacy clinical trial. AJCN. 2016;103(4):1145-1161.

S6 Bahwere P, Akomo P, Mwale M, et al. Soya, maize, and sorghum–based ready-to-use therapeutic food with amino acid is as efficacious as the standard milk and peanut paste–based formulation for the treatment of severe acute malnutrition in children: a noninferiority individually randomized controlled efficacy clinical trial in Malawi. AJCN. 2017;106(4):1100-1112.

S7 Oakley E, Reinking J, Sandige H, et al. A ready-to-use therapeutic food containing 10% milk is less effective than one with 25% milk in the treatment of severely malnourished children. J Nutr. 2010;140(12):2248-2252.
